# Elite and Sub-elite Athletes and Pregnancy: Training, Performance, Health and Psychological Aspects Across the Pre-, Peri-, and Postnatal Stages: A Scoping Review

**DOI:** 10.1186/s40798-026-01000-5

**Published:** 2026-03-09

**Authors:** Jana Nolte, Isabell Thal, Emily Büthe, Susanne Weber, Petra Platen, Kirsten Legerlotz

**Affiliations:** 1 Department of Sports Medicine and Sports Nutrition, Faculty of Sport Science, Ruhr University Bochum, Bochum, Germany; 2 Practice for Gynecology and Obstetrics, specialized in Sports and Exercise Gynecology, Heidelberg, Schriesheim, Germany; 3 Department of Movement and Training Sciences, Institute of Sport Science, University of Wuppertal, Wuppertal, Germany

**Keywords:** Gestation, Perinatal health, High-performance athletes, Olympic-level athletes, Female research, Hormones

## Abstract

**Background:**

The number of elite female athletes navigating pregnancy continues to rise, yet the intersection of high-performance sport and motherhood remains understudied. This scoping review summarizes the literature on training, performance, physical health, and psychological aspects before, during, and after pregnancy in elite and sub-elite athletes (tiers 3–5). The aim is to identify knowledge gaps and to inform future research.

**Methods:**

This review was conducted in accordance with the PRISMA-ScR guidelines and was registered with PROSPERO (CRD420250651470). At 8th of January 2025, a systematic search of 10 databases (e.g., PubMed, Scopus, and PsycINFO) was conducted. Studies were eligible for inclusion if they involved highly (or more) trained female athletes during the pre-pregnancy, pregnancy, or postpartum phases. Data extraction included information on study design, athlete classification, training, health, performance, and psychological outcomes.

**Results:**

Of the 5236 records examined, 101 studies met the inclusion criteria and 46 original research articles underwent detailed data extraction. Elite and sub-elite athletes often plan their pregnancies very carefully. The available evidence does not clearly demonstrate negative effects of high training loads on pregnancy outcomes. However, the limited, often outdated, and predominantly endurance-focused data do not allow firm conclusions. Evidence shows that elite and sub-elite athletes typically continue to train throughout pregnancy, adjusting the load, and resume training early after childbirth. Although highly individualized, performance recovery is feasible. Moderate-intensity exercise appears to be safe, but thresholds above 90% of maximum heart rate may impact fetal responses. Psychological stress, identity conflicts, and a lack of tailored guidelines are common challenges. Most birth outcomes match or exceed those of the general population.

**Conclusions:**

While no consistent evidence of adverse pregnancy outcomes from high training loads has been reported, the existing studies are too limited and heterogeneous to allow firm conclusions. These gaps, along with an evidence base largely derived from endurance-focused sports and Western populations, highlight the ongoing need for more diverse, contemporary, and sport-specific research on training, return-to-sport, and mental health in pregnant elite athletes.

*Registration* The protocol for this review was registered in the PROSPERO database (CRD420250651470).

**Supplementary Information:**

The online version contains supplementary material available at 10.1186/s40798-026-01000-5.

## Key points

- Athletes are able to safely continue training during pregnancy and subsequently return to elite-level performance after childbirth. This necessitates individualized adaptations to training regimens and careful consideration of the athlete’s specific physiological and psychological changes during pregnancy and the postpartum period.
- Future studies should categorize different sports into groups in order to develop specific training, postpartum recovery, and psychological support guidelines for elite athletes in each group.
- Future studies should address underrepresented sport populations, employ prospective designs, and incorporate interdisciplinary care models.

**Supplementary Information:**

The online version contains supplementary material available at 10.1186/s40798-026-01000-5.

## Background

The health benefits of exercise during pregnancy are widely acknowledged, including improved cardiovascular fitness, enhanced mental well-being, and positive effects on maternal and fetal health [1–5]. These guidelines advise pregnant individuals to engage in regular moderate-intensity exercise and emphasize safety considerations such as avoiding supine positions after the first trimester, monitoring exertion, and individualizing training based on symptoms. However, these and other general guidelines are designed for the general population and do not account for unique challenges resulting from the high training load and other possible risks associated with training, highlighting a critical gap this review aims to address [6–10]. The participation of women in competitive sports continues to grow, so does the number of elite athletes who become pregnant. According to the Patient Rights Act, they have a right to comprehensive and evidence-based health information [11]. However, the evidence base for the current recommendations appears insufficient. Therefore, a fundamental step is to gain a deeper understanding of pregnancy-related changes in musculoskeletal and functional characteristics, to improve and strengthen evidence-based information on safe and healthy exercise during pregnancy.

The psychological implications of pregnancy and motherhood for elite athletes are significant, beyond the physical aspects. The transition to motherhood can provoke heightened stress and anxiety, as well as shifts in identity, particularly in relation to athletic performance and career expectations [12, 13]. It is crucial to address these psychological dimensions in order to develop multidisciplinary support systems that enable a balanced transition without compromising athletic careers [14].

Returning to elite performance after childbirth presents further challenges, including physical recovery, managing mental health, and navigating new social dynamics [8, 13]. It is essential to understand these complex transitions in order to devise strategies that support elite athletes in regaining competitive form while managing the demands of motherhood.

This scoping review systematically maps the current evidence on training, performance, physical health (including birth outcomes), and psychological aspects among sub-elite and elite female athletes throughout the pre-, peri-, and post-pregnancy periods. Unlike previous reviews, this review adopts a focused inclusion strategy that considers all types of original research and review articles involving elite and sub-elite athletes classified as tier 3 (highly trained/national), tier 4 (elite/international) tier 5 (world class) [15]. This narrow focus enables a more precise understanding of this specific and understudied population. In doing so, the review not only incorporates newly published studies (up to 2025) that were not addressed in earlier syntheses, while also identifying relevant evidence that was previously overlooked. By applying rigorous and clearly defined eligibility criteria, this review provides a robust, targeted evidence base for aspects of training, performance, physical health, and psychological aspects throughout the entire continuum of pre-, peri- and postnatal stages. Our aim is to highlight persistent research gaps to inform future empirical studies.

## Methods

**Fig. 1:**
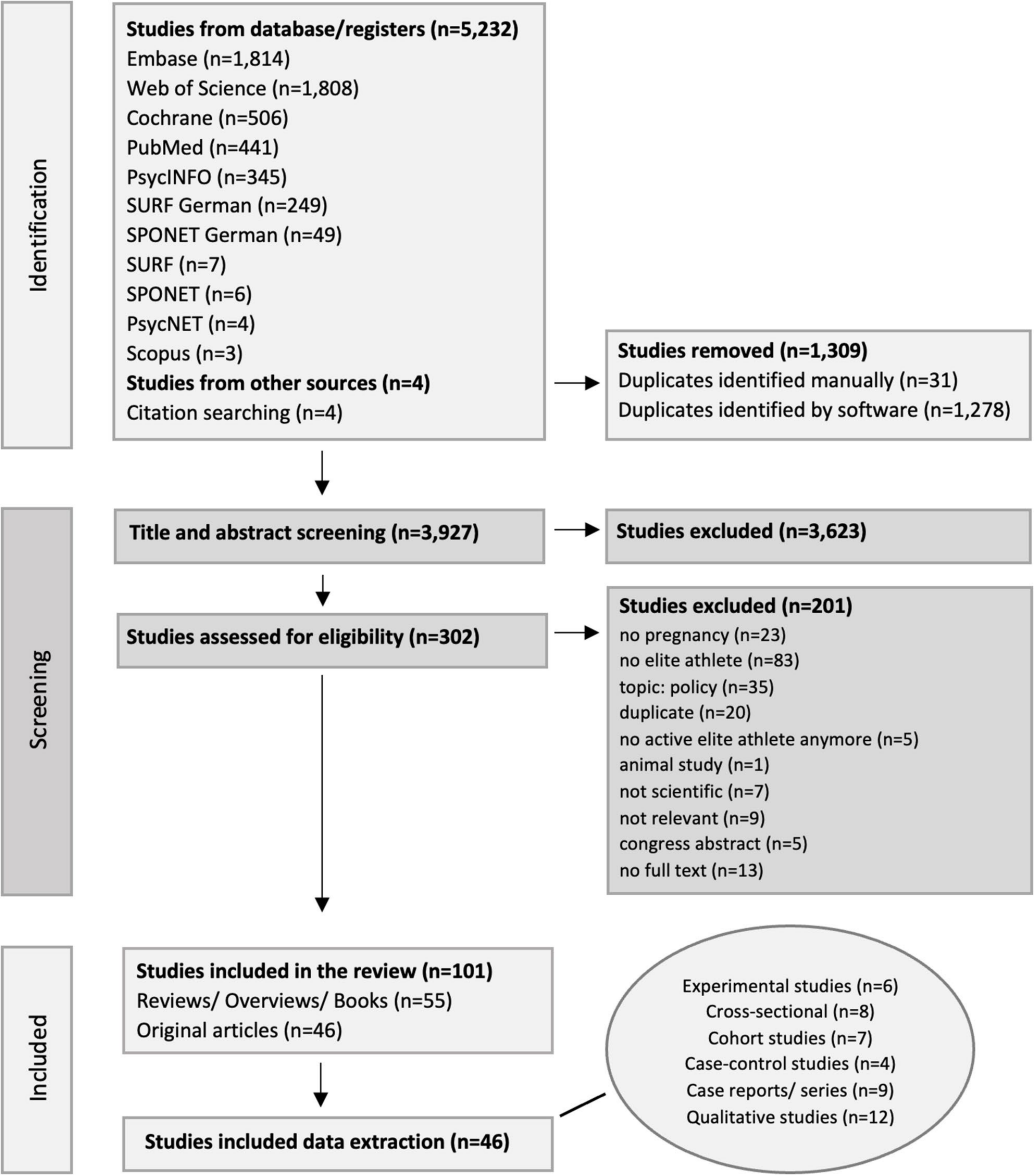
Flow chart of the study selection process (8–29 January 2025).

This scoping review was conducted and reported in accordance with the Preferred Reporting Items for Systematic Reviews and Meta-Analyses extension for Scoping Reviews (PRISMA-ScR) guidelines [16]. The review protocol was registered with the PROSPERO database (CRD420250651470). Figure 1 presents the flow chart of the study selection process.

### Eligibility Criteria

The eligibility criteria for the literature included in this review were guided by the CoCoPopS framework [17] to identify appropriate studies. *Population.* The population of interest was elite and sub-elite female athletes classified as tiers 3–5 [15], who were highly trained and performed at a world-class level, with a minimum of eight hours of training per week. Athletes who appeared to belong to tier 3, but were not explicitly assigned to a classification in the article, were categorized based on other indicators (Appendix A1) and labeled with an asterisk (*). Inconclusive study groups comprising a significant proportion of elite female athletes identified by terms such as “competitive” or “professional” were also included in the analysis and labeled as a *mixed group*. *Condition.* The review focuses on the training of highly trained athletes before, during, and after pregnancy. The focus is on understanding the key factors athletes need to consider when becoming pregnant, training during pregnancy without endangering themselves or their unborn child, and returning to their optimal performance level after childbirth. These factors include training, performance, physical health, and psychological aspects. *Context.* Studies were eligible for inclusion if they reported on athletes at any point before, during, or after pregnancy and if the athletes were active in a high-performance sports environment at that time. No predefined time limits before or after pregnancy were applied. Instead, studies were included based on their relevance to the pre-, peri-, or postpartum period, and all samples consisted of women within reproductive age. To capture all relevant original studies, reports, and reviews on the topic, primary research studies were eligible for inclusion, irrespective of their design. The focus was placed on original studies, encompassing randomized controlled trials, controlled trials, single-case, observational studies, and qualitative studies. Reviews, overviews and books or book chapters were also included to identify original studies that might have been overlooked during the initial search.

### Search Strategy and Study Selection

To address the review questions, a preliminary search was conducted in January (2025), without restricting the year of publication. A structured search was then carried out across multiple electronic databases, including PubMed, MEDLINE, Cochrane, Web of Science, Scopus, PsycNet, EMBASE, PsycINFO, Sponet, and Surf. A sensitive search strategy was applied, incorporating free text terms pertinent to the research, and, where possible, focusing on titles and abstracts related to elite athletes and pregnancy. The full search strategy is detailed in Appendix (A2).

The study selection process involved two independent raters (IT and JN), who screened the titles and abstracts of the identified studies to determine their relevance. In cases of uncertainty, the full text of the articles was retrieved for further evaluation. In the second step, the same raters assessed the full-text articles independently for final inclusion. Any discrepancies were resolved through discussion or by consulting a third or fourth reviewer (PP or KL). Additional articles were retrieved from the reference lists of included studies where applicable.

### Data Extraction

Formal data extraction was performed for all the original articles included in the study (Table 1). Two raters (IT and JN) extracted the relevant data independently. The following data were extracted: author, year of publication, study design, population, context, condition, and outcome, along with the major findings. To provide a comprehensive understanding of the experience of athletes, the results were organized within a temporal framework comprising three main phases: before pregnancy, during pregnancy, and after pregnancy. Within each phase, the findings were categorized thematically into four domains: training, performance, physical health, and psychological aspects. Further, the findings on birth outcomes were summarized. The main outcomes were training and performance parameters, competitions, maternal and fetal heart rate, body weight, complications, symptoms, injuries, return to sport and competition, breastfeeding, fertility, safety concerns, advice, anxiety, and identity. If available, birth outcomes were documented as follows: birth weight, birth mode, onset of labor, length of labor, Apgar score, and complications.

**Table 1:** Extracted data from the 46 original articles related to pre-, peri- and postnatal stages in elite and sub-elite sports. The studies are listed in alphabetical order by the name of the first author

Reference	Study design	Population	Context	Condition	Outcome
Appleby and Fisher [58]	Qualitative study (semi-structured interviews)	*n*=10 Tier 3-5 Distance running Age: ~35.6 years	After pregnancy	Psychological aspects; Training	**After pregnancy**: *Role conflict*: Conflict between two responsibilities as athlete and mother early PP (e.g. cancellations); resisting social stereotypes of motherhood; roles later seen as compatible → facilitated return to training/competition. *Coping strategies*: Recognized running as an important aspect of life; reframed as enjoyment, stress relief, personal time, not just training. *Identity*: A new athletic identity; shift from performance-based identity to multidimensional self, including motherhood. *Emotional chances*: Increase in life quality and performance; less pressure, stronger role model function → improved well-being and broader self-image.
Beilock et al. ([29]	Cross-sectional study	*n*=26 Mixed group Swimming, track and field, road racing Age: ~30.7 years	During and after pregnancy; Birth outcomes	Training; Performance	**During pregnancy**: *Training participation*: 89% trained in first Tri, 65% in third → overall reduced effort. *Training intensity management*: 42.3%: HR <140; 38.5%: listened to body; 30.8%: maintained training; 11.5%: other/none. **Birth outcomes**: *Birth weight*: Ranged from 2268 g to 4536 g; Higher exercise intensity → increased birth weight. *Fetal complications*: More strength training → fewer complications. **After pregnancy**: *Training paticipation*: Continued >6 wks PP. *Return to sport/ competition*: 81%: returned to prepregnancy fitness/ body mass; 46%: training; 54%: competition; 35%: improved personal best+F16; 8%: win (vs. 27% pre-pregnancy). *Barriers to training*: Lack of energy (pregnancy/PP); lack of time (main barrier). *Self-efficacy*: Higher during pregnancy → more effort, faster progress, earlier return, more perceived success.
Bianchedi et al. [41]	Cohort study	*n*=55 Tier 4-5 Skill sports, endurance, power, etc. Age: ~31.0 years	During and after pregnancy; Birth outcomes	Training; Physical health; Performance	**During pregnancy**: *Body mass*: +~13.8 kg (range: 5–30 kg). *Sleep*: Sleep duration increased. *Urogenital ailments*: None in 96.4%; no pre-eclampsia cases. *Injury*: 98.2% reported no injuries. *Training paticipation*: Stable until late second Tri, then declined. *Competitions*: Stopped by late first/early second Tri. **Birth outcomes**: *Birth mode*: 71.2% spontaneous, 7.7% induced, 21.2% cesarean. *Apgar score*: 9.8. *Birth weight*: ~3.2 kg. *Birth size*: ~51 cm. **After pregnancy**: *Breastfeeding*: 63% no difficulties; 60% used support; 34.5% stored milk. *Training paticipation*: Increased after 3 months; began with lower limb/abdominal work → walking/stretching → more aerobic/anaerobic training. *Return to competition*: ~7 months avg. (1–36 months); first competition ~1 year PP after body mass stabilized; 50.6% returned to international level.
Bø & Backe-Hansen [44]	Cross-sectional study	*n*=77 (*n*=31 elite athlete, *n*= 46 age-matched women from the general population) Tier 3 Different sports Age: ~34.0 years	After pregnancy; Birth outcomes	Training; Physical health; Performance	**After pregnancy**: *Training volume*: 14 h/wk prepregnancy; 4 h at 6 wks PP; 10.8 h at questionnaire. *Training mode*: More strength training (abs/back) during pregnancy & early PP in EA; 38% of EA started jogging vs. 4.3% in control group. *Symptoms*: No significant differences in low back pain, sciatica, or pelvic girdle pain. *Urinary incontinence*: 39% (EA), 37% (control); mainly stress incontinence. *Body mass*: Lower BMI at 6 wks PP and at questionnaire in EA; more regained prepregnancy weight. *Return to competition*: 77% continued at same level. **Birth outcomes**: *Birth weight*: No differences (only lower birthweight in twins (EA)); EA Ø 3290,6 g. *Onset of labor*: 45.2% at term, 38.7% before the due date, 16.1% after the due date. *Birth mode*: No differences. *Nursing*: 90% nursing in both groups; no significant difference in duration.
Brevik-Persson et al. [45]	Cross-sectional study	*n*=15 (*n*=15 elite athlete, *n*=15 non-pregnant elite and recreational athletes) Mixed group Different sports Age: ~31.0 years	During pregnancy	Training; Physical health	**During pregnancy**: Compared to non pregnant athletes core body temperature (temp) measured during high-intensity running at ~29 wks (range: 25-35 wks) was slightly higher at baseline before training; higher peak skin temp and maternal HR during training; slower core temp decline in 20-min recovery; greater fluid loss (absolute/relative); lower thermal sensation ratings. → Core temp increased during training, similar between groups (p: 1.19 °C vs. np: 1.22 °C).
Bung et al. [38] *Dataset duplicate A*	Case report	*n*=1 Tier 3* 400 m Age: 25 years	During and after pregnancy; Birth outcomes	Training; Physical health; Performance	**During pregnancy**: *Body mass*: +9 kg. *Maternal HR*: Increased at rest over pregnancy. *Fetal HR*: Increased during exercise; bradycardia after sprints. *Uterine activity*: Unchanged during/after strenuous exercise. *Symptoms*: Dizziness after sprints. **Birth outcomes**: *Birth weight*: 3200 g. *Onset of labor*: 11 days post due date. *Apgar score*: 9 & 10. **After pregnancy**: *Performance*: New personal records <6 months PP. *Maternal HR*: Peak HR during submax. exercise in puerperium; lowest at 6 months PP.
Bung et al. [37] *Dataset duplicate A*	Case-control study	*n*=22 (*n*=1 elite athlete, *n*=21 pregnant woman) Tier 3* 400 m Age: 25 years	During and after pregnancy; Birth outcomes	Training; Physical health; Performance	**During pregnancy**: *Body mass*: +9 kg. *Maternal HR*: Lower at rest and during exercise in athlete vs. control. *Fetal HR*: Lower at rest and post-exercise in athlete. *Blood pressure*: Stable in athlete; increased in control; during exercise lower in athlete. *Respiratory rate & minute volume*: Lower in athlete at rest and during exercise; max. values at wk 36 in both groups. *O2 uptake*: Lower in athlete at rest and during exercise. *Vital capacity*: No group difference. *FEV1*: Higher absolute values in athlete. **Birth outcomes**: *Birth weight*: 3220 g. *Onset of labor*: 11 days after due date. **After pregnancy**: *Performance*: Lowest at 11 days PP, highest at 6 months PP (athlete). *Maternal HR*: Highest 11 days PP; lowest at 6 months. *Lactate*: Lowest at 11 days, highest at 6 months PP → aligned with performance. *O2 uptake*: Highest at 11 days PP. *Vital capacity*: Lower in athlete at 6 months PP.
Cohen et al. [46]	Case series	*n*=2 Tier 3* Running Age: 28 (case 1); 34 (case 2)	During pregnancy; Birth outcomes	Training; Physical health	**During pregnancy**: Case 1 – *Training volume (m/wk)*: 50 prepregnancy; 54.4 (Tri 1), 34 (Tri 2), 13 m/wk (Tri 3). *Body mass*: +19 kg. *Blood pressure*: No change. *Fetal HR*: Normal. *Symptoms*: Mild preeclampsia diagnosed in Tri 3. Case 2 – *Training volume (m/wk)*: 35–40 prepregnancy; 42 (Tri 1), 28 (Tri 2), 12 m/wk (Tri 3). *Body mass*: +8.1 kg. *Blood pressure*: Not changed. *Fetal HR*: Normal. **Birth outcomes**: Case 1 – *Onset of labor*: 40 wks. *Labor length*: 18 h. *Birth mode*: cesarean section. *Birth weight*: 3970 g. Case 2 – *Onset of labor*: 40 wks. *Labor length*: 39 h. *Birth mode*: vaginal delivery. *Birth weight*: 3400 g.
Darroch et al. [22]	Cohort study	*n*=42 Tier 4-5 Running Age: ~31.7 years	During and after pregnancy	Training; Physical health; Performance	**During pregnancy**: *Training volume*: 57% conceived with reduced training; 24% reduced training to aid conception. Prepregnancy: 9 ± 2 sessions/wk → volume decreased significantly during pregnancy, but sessions/wk remained at ~5–6 ± 2. *Training intensity*: Pace was significantly slower than prepregnancy across Tri 1-3; no change in number of low-intensity runs; moderate-/high-intensity runs declined. **After pregnancy**: *Training volume*: Avg. 6 ± 6 wks off running and 3 ± 2 off cross-training; ~80% of prepregnancy load reached by 14 ± 11 wks. *Training intensity*: Fewer low-intensity runs than prepregnancy. *Performance*: Race participation lower up to 5 years. Among those sought to return to previous level, no performance drop in years 1–3; 56% improved. In those not intending to return, 4.6% decline. High performers had higher training volumes in Tri 1 (73%) and Tri 2 (54%) vs. lower performers (64%/49%); Tri 3 volume was significantly higher in those who matched/exceeded prior performance. *Injury*: 50% reported PP injuries: 6 bone stress (incl. 2 sacral), 11 musculoskeletal (muscle/tendon/ligament), 2 nerve-related (sciatica), 2 “other”.
Davenport [56] *Dataset duplicate B*	Qualitative study (semi-structured interviews)	*n*=18 Tier 3-5 Olympic sports Age: ~35.0 years	After pregnancy	Training; Physical health; Psychological aspects	**After pregnancy**: *Physical challenges*: Fatigue, uncertainty; physical changes affected performance; fear of returning too soon; mixed experiences (some struggled, others improved). *Emotional challenges*: Frustration, some experienced mental health issues, including PP depression. *Injuries*: Common: pelvic organ prolapse, stress fractures, SI joint pain, abdominal strain. *Ressources*: need for better support/ resources. *Breastfeeding*: Difficult to coordinate; training better after feeding; concerns over milk supply. *Role conflict*: Athletes were able to do both but faced external expectations; pressure to perform to remain on teams; required personal adjustment.
Davenport et al. [23] *Dataset duplicate B*	Qualitative study (semi-structured interviews)	*n*=20 Tier 4-5 Olympic sports Age: ~35.0 years	Before and during pregnancy	Training; Physical health; Psychological aspects	**Before pregnancy**: *Role conflict*: Societal pressure to choose between sport and motherhood. *Resources*: Support from other pregnant elite athletes was key. *Emotional challenges*: Fear of career/financial loss; tension between reducing training for fertility vs. maintaining performance; fear of disclosing pregnancy → risk of losing team spot or being seen as less committed; fear about acceptance. *Planning*: Desire to conceive post-Olympics; financial factors influenced timing. **During pregnancy**: *Physical and emotional challenges*: Internal/ external expectations clashed with reality → Frustration, uncertainty; fear training could harm health or fertility; uncertainty due to limited research on elite athletes. *Resouces*: lack of evidence-based guidance.
Davies et al. [39]	Case report	*n*=1 Tier 3* Marathon running Twin pregnancy Age: 33 years	During pregnancy; Birth outcomes	Training; Physical health	**During pregnancy**: *Body mass*: +9.7 kg from wk 4 to 32 (steady gain). *Blood pressure*: Stable. *Resting supine HR*: Increased from 48 to 57 bpm (wk 1–36). *Hemoglobin concentration*: 12.2 g/dL (wk 8) → 12.6 g/dL (wk 35). *Obstetric cholestasis*: Diagnosed 3 days pre-birth (bilirubin 24 → 33 µmol/L). *Training volume and intensity*: Prepregnancy: 155 km/wk, HR 140–180; during pregnancy: 107 km/wk, HR 130–140. *Submaximal treadmill test*: At wk 29: HR, La, VO₂, RPE all higher vs. 10 wks PP → elevated metabolic/cardiorespiratory load during pregnancy. **Birth outcomes**: *Birth mode*: Elective cesarean at 36 wks.
Diggles [57]	Case report	*n*=1 Tier 3 Netball Age: 27 years	After pregnancy	Physical health; Psychological aspects	**After pregnancy (+pelvic floor programm)**: *Psychological outcomes*: Low risk of anxiety/depression. Fear of movement and fatigue peaked at 4 wks, declined thereafter. Readiness to return to sport increased steadily, highest at 6 months. *Levator ani tone*: Normal throughout. *Levator ani strength*: Partial relaxation at 4 wks; strong/normal relaxation at 8 wks and 6 months. *Pelvic floor muscle endurance*: Marked increase from 4 to 8 wks. *Pelvic floor muscle speed*: Weakest at 4 wks (delayed response); strong improvement after 8 wks. *Rectus abdominis strength*: Lowest at 4 wks; highest at 6 months. *Musculoskeletal outcomes*: Improved from 4 to 8 wks.
El-Bsat [51]	Controlled Intervention	*n*=48 (*n*=24 intervention group, *n*=24 control group) Mixed group Basketball and volleyball Age: 20-30 years	During pregnancy	Psychological aspects	**During pregnancy**: *Anxiety and depression levels*: Significantly less in experimental group vs. control (HDA scale) at 30 wks. *Anxiety scores*: Experimental: 7.29 → 3.83; control: 7.50 → 5.21. *Depression scores*: Experimental: 8.17 → 1.92; control: 8.25 → 4.75. → Psycho-pedagogical interventions led to significant reduction in anxiety and depression.
Forstmann et al. [53]	Cohort study	*n*=37 (compared to IMAP1 model) Tier 4 Marathon running Age: ~28.4 years	After pregnancy	Training; Performance	**After pregnancy**: *Performance*: 70.28% achieved personal best after pregnancy. *Return to sports*: Range: 9–94 months; median: 23 ± 11 months. No differences in performance development considering age and one or two pregnancies. → Return to sport and performance improvement primarily depend on age at pregnancy relative to peak performance age.
Franklin et al. [30]	Cross-sectional study	*n*=224 (different activity levels) Mixed group Rowing Age: ~36.0 years	During and after pregnancy	Training; Physical health	**During pregnancy**: *Training paticipation*: 85.2% (98/115) of previously pregnant rowers trained during pregnancy. *Training volume*: 51.3% (Tri 1), 42.4% (Tri 2), 15.7% (Tri 3). → Rowers struggled to meet guidelines (>150 min/wk), similar to the general population. A comparable number exceeded guidelines via ≥150 min/wk vigorous+ intensity: 26.1% (Tri 1), 11.3% (Tri 2). *Competition paticipation*: 31.3% competed in a regatta; 35.7% in other sporting events. *Safety concerns*: Concerns included abdominal trauma from the oar, high-intensity training risks, team-related guilt, and mental strain from performance loss. Concerns rose with gestational age, especially around high-intensity training/ competition. Younger, less experienced, and nulliparous rowers perceived more risk. 34.6% reported no barriers. *Symptoms*: Fatigue, nausea. *Advice*: Primary sources: healthcare providers (60.2%), internet (30.8%), friends (25.6%). **After pregnancy**: *Return to sport*: Most resumed training PP; participation eventually limited by physical changes (e.g., abdominal size).
Giles et al. [52]	Qualitative study (semi-structured interviews)	*n*=11 Tier 4-5 Running Age: 25-37 years	After pregnancy	Training; Physical health	**After pregnancy**: *Breastfeeding duration*: 1.5–18 months (mean: 9.73 months). *Challenges*: Interfered with training/competition; required milk pumping; fatigue from lack of sleep limited high-intensity training; delayed return to competition fitness; injury concerns during breastfeeding. *Received advice*: Limited access; sought from internet, peers, healthcare providers, elite athletes → but faced inconsistent or lacking info. *Safety concerns*: Concern for baby’s health; fear that early weaning to resume training could harm infant.
Hegaard et al. [28]	Multi-center cohort study	*n*=4718 (*n*=190 competitive, *n*=1171 moderate-to-heavy, *n*=3099 light, *n*=258 sedentary) Mixed group Different sports Age: >18 years	Before and during pregnancy	Training; Physical health	**Before pregnancy**: Competitive/moderate-to-heavy group: older, no chronic disease, 12 years education, non-smokers. **During pregnancy**: *Training participation*: In competitive group (*n* = 190) only 2% (*n* = 3) still did competitive sports, 14% (*n* = 28) moderate-to-heavy exercise, 71% (*n* = 135) light execise, 12% (*n* = 24) were sedentary. → Most active women before pregnancy stayed most active during pregnancy.
Hegaard et al. [48]	Cohort study	*n*=1827 (*n*=36 competitive, *n*=276 moderate, *n*=1298 light, *n*=217 sedentary) Mixed group Different sports Age: >18 years	During and after pregnancy; Birth outcomes	Physical health	**During pregnancy**: *Body mass*: Competitive athletes had 2.6× higher risk of exceeding IOM weight gain; ~60% gained more than recommended. **Birth outcomes**: *Birth weight*: No link between pre-pregnancy activity level and birth weight. **After pregnancy**: *Body mass*: No group differences.
Kardel [20] *Dataset duplicate C*	Controlled intervention	*n*=41 (*n*=20 high-volume (HEG), *n*=21 medium valume (MEG)) Mixed group Different sports Age: ~26.7 years	During and after pregnancy	Training	**During pregnancy vs. after pregnancy**: *Skin fold*: No differences. *Maternal resting HR*: ↑ during pregnancy, ↓ PP. *Maternal exercise HR*: No differences during pregnancy; ↓ in HEG (50/100 W) at 12 wks PP. *O₂ consumption*: Not changed (50/100 W); ↑ in HEG at 150 W (max) PP vs. pregnancy. *Lacatate*: No differences. *RPE*: No differences. → Both groups responded similarly to exercise despite differing baseline aerobic capacity.
Kardel & Kase [62] *Dataset duplicate C*	Controlled intervention	*n*=42 (*n*=20 high-volume (HEG), *n*=21 medium valume (MEG)) Mixed group Different sports Age: ~26.7 years	During and after pregnancy; Birth outcomes	Training; Physical health	**During pregnancy vs. after pregnancy**: *HR*,* body mass*,* skin fold*,* fetal growth*,* ultrasound*,* nonstress tests*: No substantial differences between the two exercise groups. **Birth outcomes**: *Onset/duration of labor (vaginal)*,* pain control*,* perinatal complications*,* 1-/5-min Apgar*,* birth weight*,* placental weight*: No substantial differences between HEG and MEG. Labor duration and birth/placental weights tended to be higher in HEG, but not statistically significant.
Kleinert & Sulprizio [21]	Qualitative study (semi-structured interviews)	*n*=3 Tier 3-4 Swimming, speed skating, climbing Age: 31-36 years	Before, during and after pregnancy	Training	**Before pregnancy**: *Training participation*: 5–8 sessions/wk, 1.5–2 h each. **During pregnancy**: *Training load*: Initially unchanged; later reduced intensity (only aerobic), lower volume, body awareness, fall risk avoidance, abdominal limitations. After 6th month: less jogging/walking, more swimming/cycling. *Received advice*: Positive support from gynecologists/midwives for high activity; 2/3 athletes relied mainly on peer support. *Symptoms*: Back pain, fatigue → reduced volume/intensity or switched to cycling/swimming. **After pregnancy**: *Training mode*: Early comeback with pelvic floor training; walking, cycling, swimming from 6–8 wks PP. *Performance*: One athlete expected performance gains (hormonal), but all prioritized family over sport.
Königstein [42]	Case report	*n*=1 Tier 4 Marathon running Age: 30 years	During and after pregnancy	Training; Performance	**During pregnancy**: *Training participation*: 1–2 anaerobic sessions/wk until month 8. Carbohydrate intake is emphasized during intense sessions. *Training planning*: No performance focus; Injury prevention through targeted pelvic floor and abdominal training during pregnancy and PP. **After pregnancy**: *Training load*: Increased gradually from wk 4. *Performance*: Improved post-breastfeeding; no performance training until month 3; near pre-pregnancy level by month 7; new records at month 8. *Return to sport*: Functional/isometric ab exercises at 1 wk; walking at 2 wks; cycling at 3 wks.
Krapf [50]	Qualitative study (semi-structured interviews)	*n*=8 (*n*=5 active; *n*=3 former) Tier 4-5 Different sports Age: 30-36 years	After pregnancy	Psychological aspects	**After pregnancy**: *Role*: Athletes frequently combine roles (athlete + mother, athlete + job + mother). Armed forces support structures helped some reduce conflicts. *Social support*: Emotional and mental support from the immediate environment as a key resource. *Challenges*: Negative judgments from others; loss of squad status due to pregnancy; difficulties re-entering elite sport after childbirth; national team coaches often perceived as hindering rather than supportive.
Kuczera et al. [26]	Case-control study	*n*=32 (*n*=16 elite athletes, *n*=16 non althete) Tier 3-5 Judo Age: ~26.3 years	Before pregnancy; Birth outcomes	Training; Physical health	Training before pregnancy and impact on labor **Birth outcomes**: No significant differences in labor induction, augmentation, delivery mode, labor duration, perineal tears, episiotomy, or Apgar scores between elite judo athletes and non-athletes → no negative impact of elite judo training on first childbirth outcomes. - Among 16 elite judo athletes: 62.5% vaginal, 31.25% intrapartum cesarean, 6.25% vacuum-assisted delivery. - Among 11 elite judo athletes: 56.25% had labor induced (oxytocin); 45.5% had perineal tears (27.3% grade I, 18.2% grade II); 45.5% had episiotomy.
Lotgering et al. [43]	Cohort study	*n*=33 Mixed group Different sports Age: ~30.9 years	During and after pregnancy	Performance	**During pregnancy**: *VO₂max*: Unchanged. *Resting O₂ uptake*: Increased → less O₂ available for exercise. *Maximum CO₂ production*: Lower; hyperventilation persisted. *Maximum HR*: Slightly decreased (~4 bpm). **After pregnancy**: *Performance*: Significantly lower at 35 wks PP
Martínez-Pascual et al. [25] *Dataset duplicate D*	Qualitative study (unstructured, semi-structured interviews and letters)	*n*=20 Tier 4 Different sports Age: ~37.5 years	Before and during pregnancy	Psychological aspects	**Before pregnancy**: *Planning of pregnancy*: Pregnancies were planned to reduce sport-related disruption; many delayed motherhood. **During pregnancy**: *Emotional challenges*: Physical activity helped athletes stay connected to their bodies, but they faced misunderstanding from others and had to justify their training; uncertainty about safe training; sought professional guidance; concerns about returning to elite sport, maintaining performance, and securing financial support.
Martinez-Pascual et al.[49] *Dataset duplicate D*	Qualitative study (unstructured, semi-structured interviews and letters)	*n*=20 Tier 4 Different sports Age: ~37.5 years	During pregnancy	Psychological aspects	**During pregnancy**: *Physical challanges*: “A new body” mixed reactions - some embraced changes, others struggled with identity; desire to regain pre-pregnancy body while accepting changes as part of motherhood; “Body control” loss of control impacted emotions and athletic identity; sports background seen as helpful for labor (body awareness, pain tolerance). *Emotional challenges*: “Feeling and communicating with the body” athletes sensed bodily signals but didn’t always respond; “Body’s beauty ideal” pressure from aesthetic and sport-specific standards; body image and weight highly relevant → pregnancy changes were hard to accept; strong wish to return to pre-pregnancy form.
Martinez-Pascual et al. [59] *Dataset duplicate D*	Qualitative study (unstructured, semi-structured interviews and letters)	*n*=20 Tier 4 Different sports Age: ~37.5 years	After pregnancy	Psychological aspects	**After pregnancy**: *Identity*: “A new identity” maternity seen as a transformation; “sportswoman” identity shifted to “mother”; visibility in sports replaced by feelings of anonymity. *Emotional challenges*: “Return to sport” mixed emotions — guilt, need to justify comeback; all reported a new, special motivation upon return; “Biggest challenge” balancing motherhood and sport; pride in becoming “sportswomen mothers. *Emotional chances*: “Reaching a goal” maternity viewed as a life goal to be fulfilled.
Massey & Whitehead [60]	Qualitative study (semi-structured interviews)	*n*=2 Tier 4-5 Olympic discus throwing and paralympic sprint	After pregnancy	Psychological aspects	**After pregnancy**: *Identity*: “Athlete identity” reduced at 2 months; regained by para-athlete after return to competition (16 months); Olympic athlete did not regain it due to loss of sponsorship.“Mother identity” experiences differed; both viewed “elite athlete mother” as a strong, dual identity, balanced positively. *Physical challenges*: Bodily changes; acceptance varied. At 6 months, bodies more athletic but not optimal; performance considered good under the circumstances. *Role conflict*: Sport was top priority before pregnancy; PP, para-athlete prioritized baby, Olympic athlete experienced conflicting priorities.
McGannon et al. [40]	Autobiographic study	*n*=1 Tier 5 Running Age: 36 years (first); 40 years (second pregnancy)	During and after pregnancy	Psychological aspects; Training; Performance	**During pregnancy**: *Training participation*: No strict plan; training adapted based on body signals. *Resources*: “Integration of family into training (best of both worlds)” support from husband; adjusted training times; focused on enjoyment over performance. **After pregnancy**: *Performance*: Elite-level performance achieved after childbirth.
Nose-Ogura et al. [47]	Cross-sectional study	*n*=328 Mixed group Different sports Age: ~29.1 years	During and after pregnancy; Birth outcomes	Training; Physical health	**During pregnancy**: *Training participatiom*: 54.6% exercised. *Perinatal complications*: Anemia (27.4%), miscarriage/preterm birth (10.7%) → no significant difference between exercise and non-exercise groups. **Birth outcomes**: *Birth mode*: Vaginal (68.3%), vacuum/forceps-assisted (17.1%), cesarean (14.6%: 9.7% emergency, 4.9% elective). **After pregnancy**: *Symptoms*: Low back pain (44.2%), urinary incontinence (39.9%), stiff shoulder/neck (36.9%), hemorrhoids (33.2%) → higher incontinence after vaginal vs. cesarean birth. *Physical functions*: 79.5% reported weakness (esp. lower limbs); 57.9% had prolonged fatigue recovery. *Received advice*: 29.6% (*n* = 53) exercised with obstetrician guidance; 16.5% (*n* = 39) received return-to-sport guidance. *Return to competition*: 87.5%; average return at 10.5 months PP → slightly earlier after cesarean; 25% returned post-weaning; >50% while breastfeeding.
O’Leary et al. [12]	Qualitative study (semi-structured interviews)	*n*=11 Tier 4-5 Different sports Age: ~31.0 years	After pregnancy	Training; Psychological aspects	**After pregnancy**: *Identity*: “Changing priorities and shifting identities” balancing athlete and mother roles was challenging. *Breastfeeding*: Difficulties due to pressure to maintain milk supply and pain during contact sports. *Health*: Common issues included injuries and urinary incontinence. *Mental readiness*: Return to sport supported mental health but brought added stress due to lack of guidance, fitness insecurity, and financial concerns. *Resources*: “Peer and family support” team, family, and medical support (e.g., childcare at training) were essential for returning. *Role conflict*: Observing other athletes’ return experiences was motivating and informative. *Received advice*: Nutrition advice was often too general.
Penttinen & Erkkola [27]	Case-control study	*n*=60 (*n*=30 elite athlete, *n*=30 non-athlete) Tier 3-5 Endurance sports Age: ~28.1 years	Before, during, and after pregnancy; Birth outcomes	Training; Physical health; Performance	**Before pregnancy**: *Menstrual cycle characteristics*: 77% regular cycles; 23% irregular (5 amenorrhea, 2 oligomenorrhea). *Complications*: 23% had prior spontaneous abortion; 5 attributed it to hard training. **During pregnancy**: *Training participation*: 23 athletes trained during pregnancy (mean: 23 wks, range 7–39); endurance training had no adverse effects. *Performance*: 53% noticed no change; 10% felt improved; 23% felt worse. *Competition participation*: 60% (*n* = 18) competed until wk 13 (range 0–25 wks). **Birth outcomes**: *Birth weight*,* labor phases*,* labor length*,* onset of labor*,* birth mode*,* injuries*: No significant differences in labor characteristics between athletes and non-athletes. **After pregnancy**: *Return to competition*: 18 athletes returned, avg. 8.2 months (range 2–24). *Performance*: 11% improved, 61% returned to same level, 28% did not regain prior level.
Perl [31]	Cross-sectional study	*n*=11 Tier 4-5 Different sports	During and after pregnancy	Training	**During pregnancy**: *Training load*: Low-intensity endurance training stayed stable early on, declined in Tri 3. High-intensity training significantly reduced; none trained >87% HRmax in Tri 3. Strength, balance, speed, jump training less frequent; core/abdominal training peaked in Tri 3. 82% avoided high intensity; contact sports and fall risk minimized. 91% preferred body awareness over strict training plans. *Competition participation*: No championships from month 4 onward. **After pregnancy**: *Training load*: Core and strength training resumed 4–6 wks PP. Some restarted high-intensity training by 6–8 wks; 36% (*n*=4) within 3 months. 70% resumed low-intensity endurance within 1 month. Return to pre-pregnancy volume varied (some within 6–9 months). One with cesarean and delayed return; three had complications (perineal tear, rectal diastasis, stress incontinence) limiting exercise.
Potteiger et al. [54]	Case report	*n*=1 Tier ≥3* Marathon Age: 34 years	After pregnancy	Training; Physical health	**After pregnancy**: *Body mass and fat*: Decreased from wk 4 to 16. *VO₂max*: Not changed over 16 wks. *VO₂-LT and VO₂-OBLA*: Increased from wk 4 to 8. *Energy expenditure*: Not changed. *Training volume*: Ranged from 50 km (wk 1) to 98 km/wk (wk 14); no double sessions.
Richard et al. [55]	Case study	*n*=1 Tier 3 Field hockey (Sprint interval training (SIT) in PP) Age: 35 years	After pregnancy	Training	**After pregnancy**: *Sprint interval training (SIT) in PPMedical clearance*: At 5 wks PP. *Cardiac output*: Maintained elevated levels from pregnancy. *VO₂peak*: Returned to baseline by 11 wks; relative V̇O₂peak remained below baseline at all points. *Peak power output*: Initially reduced, returned to pre-pregnancy levels mid-/end-intervention. *Haematological values*: Blood volume and haemoglobin mass decreased mid-intervention, recovered post-intervention. Haematocrit and haemoglobin concentration increased at all PP time points. → SIT was feasible, well-tolerated, and supported rapid return to pre-pregnancy fitness.
Salvesen et al. [35]	Experimental study	*n*=6 Tier 5 Endurance sports Age: 28-37 years	During pregnancy; Birth outcomes	Training; Physical health	**During pregnancy**: *Strenuous treadmill running at 60–90% VO₂max) during Tri 2 (23–29 wks gestation)* *Fetal HR*: Normal (110–160 bpm) when maternal HR <90% max. At >90% HR and >50% drop in uterine artery flow → fetal bradycardia and high umbilical artery pulsatility index; resolved quickly post-exercise. → Strenuous exercise >90% HRmax may compromise fetal well-being; moderate intensity is safe. **Birth outcomes**: *Onset of labor*: 36 and 42 wks. *Birth weight*: 3000–3440 g; within lower-normal range for gestational age (86–103%). One case of HELLP syndrome at 35 wks → induced labor; healthy vaginal delivery, birth weight 2285 g.
Sigurdardottir et al. [61]	Case-control study	*n*=254 (*n*=89 high impact sports (HIP), *n*=47 low impact sports (LIP), *n*=118 non-athlete) Tier 3 Different sports Age: ~26-27 years	Birth outcomes	Training; Physical health	**Birth outcomes**: *Birth mode*: No significant differences in emergency cesareans (Non-athletes: 9%, LIP: 5%, HIP: 11%); 1 elective cesarean per group. *Length of labor*: No significant differences in stage 1 (~600 min) or stage 2 (~56–65 min) across groups. *Perineal tears*: Higher in LIP athletes (23.7%) vs. HIP athletes (5.1%); no significant difference between athletes and non-athletes (12%). → Impact of training frequency: No influence on labor outcomes; no increased complication risk for elite athletes. → General conclusion: Elite sport does not negatively affect childbirth. HIP athletes may have lower risk of severe perineal tears. Safe participation with appropriate pelvic floor training.
Solli & Sandbakk [31]	Case study	*n*=1 Tier 5 Cross county skiing Age: 35 years	During and after pregnancy	Training; Physical health	**During pregnancy**: *Training volume (h/wk)*: Tri 1: 12.9 ± 7.3 (10.0); Tri 2: 18.3 ± 2.9 (18.0); Tri 3: 8.8 ± 4.4 (9.6); *Low intensity training (h/wk)*: Tri 1: 10.9; Tri 2: 15.2; Tri 3: 7.6. *Moderate intensity training (h/wk)*: Tri 1: 0.4; Tri 2: 1.3; Tri 3: 0.7. *High intensity training (h/wk)*: 2.2 total during pregnancy. *Running during pregnancy*: Normal in Tri 1 and Tri 2; reduced in Tri 3. **After pregnancy**: *Training volume (h/wk)*: Wk 1-6: 6.6; Wk 7-12: 14.1; Wk 13-18-: 10.6; Wk 19-24: 13.6 → pre-pregnancy volume regained 19 wk post-PP. *Health*: Sacrum fractures in PP → reduction in running, MIT, and HIT. *VO₂ at LT*: Dropped to 90% in Tri 3, recovered to 100% in wk 13-18. *Body composition*: PP ↑weight, ↑%fat, ↓bone mineral density, ↓lean mass → returned to pre-pregnancy levels by CP (wk 54).
Sundgot-Borgen et al. [10]	Cross-sectional study	*n*=68 (*n*=34 elite athlete, *n*=34 active controls) Tier 4-5 Different sports Age: ~33.1 years	Before, during and after pregnancy; Birth outcomes	Training; Physical health; Performance	**Before pregnancy**: No significant differences in fertility. **During pregnancy**: *Training volume*: Both groups reduced volume across Tri 1-3 and early PP. *Injuries*: 12% (*n*=4) of athletes had training-related injuries in Tri 3 (2 muscle strains, 1 fracture, 1 ankle sprain). *Symptoms*: Elite athletes reported fewer common pregnancy complaints than controls. **Birth outcomes**: No differences in complications during pregnancy and delivery. **After pregnancy**: *Training participation*: 71% of elite athletes resumed within 0–6 wks vs. 32% of controls. *Training volume*: Increased progressively. *Injury*: Only elite athletes had PP stress fractures (12%). *Urinary incontinence*: No significant differences PP. *Body image & eating disorders*: Athletes had greater body dissatisfaction and drive for thinness; clinical ED cases decreased PP in athletes, remained stable in controls. *Challenges*: Dissatisfaction with guidance on strength training/nutrition; most did not bring baby to training/competition; lack of recovery time was a major concern. *Performance*: 44% returned to the same level, 15% improved, 26% worse, 15% unsure (mean return at 4 months); most controls (82%) reported the same performance, 6% better, 12% worse. → Conclusion: Elite athletes can train safely during pregnancy and return early PP. Stress fractures may occur with too-rapid return. Better support in nutrition, strength training, and recovery is needed.
Szymanski & Satin [34] *Dataset duplicate E*	Experimental study	*n*=45 (*n*=15 highly active, *n*=15 regularly active, *n*=15 non-exercisers) Mixed group Running Age: ~33.4 years	During pregnancy; Birth outcomes	Training; Physical health	**During pregnancy**: *Moderate-intensity exercise session (all groups)*: *Fetal HR*: Increased with exercise; significant only in regularly active group. *Umbilical artery doppler indices*: No differences across groups or with exercise. *Biophysical profile score (within 30 min)*: 8/8 in both exercise groups; 14/15 in non-exercise group. *Fetal HR post exercise*: All subjects met reactivity criteria within 20 min. *Vigorous-intensity exercise session (only exercise groups)*. *Fetal HR during exercise*: Normal range; no group difference. *Fetal HR postexercise*: increased significantly in both groups. *Umbilical artery indices*: No group difference; significant time effect; no group-by-time interaction. *Biophysical profile scores*: 8/8 in all regularly active, 12/13 in highly active group. F*et al. HR post exercise*: All met reactivity criteria within 20 min. **Birth outcomes**: *Birth mode*: No group difference; all term deliveries except 1 (highly active) and 1 (non-exerciser). *Birth weight*: Similar across groups; slightly lower in highly active group (1 SGA case), 1 LGA case in both other groups. *Apgar scores*: All >7; no group differences.
Szymanski & Satin ([36] *Dataset duplicate E*	Experimental study	*n*=45 (*n*=15 highly active, *n*=15 regularly active, *n*=15 non-exercisers) Mixed group Running Age: ~33.4 years	During pregnancy	Training; Physical health	**During pregnancy**: *Threadmill test to exhaustion*: Subgroup of 5 women from the highly active group showed: *Fetal HR*: Transient fetal heart rate decelerations immediately post-exercise (~2.5 min on average). *Umbilical artery*: Higher systolic/diastolic ratio, resistance index, and pulsatility index. *Uterine artery*: Elevated pulsatility index (up to 90th percentile). *Biophysical profile (BPP)*: 8/8 within 30 min. Results from Szymanski & Satin (2012a)
Tekavc et al. [13]	Qualitative study (semi-structured interviews)	*n*=8 Tier 4-5 Different sports Age: 30-36 years	Before, during and after pregnancy	Psychological aspects; Training; Physical health; Performance	**Before pregnancy**: *Planning of pregnancy*: 7 of 8 planned pregnancy immediately after the Olympic Games. **During pregnancy**: *Physical challanges*: “The changing pregnant body” no medical issues; enjoyed physical changes and used time for rest and injury recovery. *Training participation*: Gradually decreased; sport-specific training (e.g., judo, taekwondo) stopped earlier, depending on sport. *Identity*: Continued to identify primarily as athletes. **After pregnancy**: *Emotional challenges*: Positive outlook, but some regret and uncertainty about regaining former performance. *Performance*: Declines in endurance, strength, and balance due to joint/tendon looseness and less recovery time. *Symptoms*: Pain (varied from minimal to limiting pain). *Body mass*: Weight decreased, but dissatisfaction with body shape remained. *Training load*: Started with pelvic floor and trunk stabilization; intensity increased gradually. *Resources*: Support of family. *Performance*: Technical no loss attributed to pregnancy. *Emotioal chances*: Strong passion, Olympic goals, desire to share experience with family, aim to reach or exceed prior level or retire successfully. *Priorities*: Family became central; less willingness to invest fully in sport.
Tenforde et al. [33]	Cross-sectional study	*n*=110 (comparison to those athletes who did not run during pregnancy (*n*=77) and after pregnancy (*n*=33)) Mixed group (1/3 > Tier 3) Running Age: ~44.3 years	During and after pregnancy	Training; Physical health	**During pregnancy**: *Training participation*: 70% ran at some point; one-third ran into Tri 3. Runners trained more prepregnancy. *Training intensity*: reduced to ~50% of prepregnancy level. *Injuries*: 3.9% reported running-related injuries. *Reasons to run*: Stay in shape (89.6%), maternal health (80.5%), routine (71.4%), control (37.7%), fetal health (29.9%). *Reasons to not run*: Not feeling well (54.6%), doctor’s advice (27.3%), fear of miscarriage (21.2%). **After pregnancy**: *Breastfeeding and training*: 84.1% continued running while breastfeeding. Running during breastfeeding linked to lower PP depression (6.7% vs. 23.5%). No significant impact on breastfeeding ability.
Zaharieva [24]	Cohort study	*n*=150 (olympic athletes *n*=27 vs. “masters of sport” (high-level non-olympians) *n*=59 vs. first-grade athletes (lower elite level) *n*=64) Mixed group Different sports Age: 21-25 years	Before, during and after pregnancy; Birth outcomes	Training; Physical health; Performance	**Before pregnancy**: *Fertility*: No negative effects of Olympic participation on reproductive health. **During pregnancy**: *Training paticipation*: Some continued training/competing early in pregnancy; Olympic athletes trained less than lower-tier athletes. *Complications*: 70.4% normal pregnancies; 29.6% with disturbances; Training-related complications (pain, bleeding) more common in non-Olympic high-level athletes. **Birth outcomes**: *Birth weight*: 3100–3500 g. *Birth mode*: 81.4% normal deliveries among Olympians; 18.6% required accelerated delivery; similar in other groups. First stage of labor longer, expulsive stage shorter. **After pregnancy**: 70.4% of Olympians had normal PP periods. *Breastfeeding*: Unaffected in most (48.2%); 18.5% reported reduced lactation due to training. *Return to sport*: Most returned to training by 3–5 months PP. *Return to competition*: Later in Olympians vs. others but often matched or exceeded pre-pregnancy performance. *Complications*: Highest in “masters of sport” group, likely from inadequate training. *Performance*: Olympians reported feeling physically stronger and more balanced PP.

*An asterisk marks the samples for which the classification could only be assigned speculatively based on secondary criteria (see Appendix A1)

Trimester (Tri), postpartum (PP), heart rate (HR); week (wk) Dataset duplicate describes that several studies refer to the same dataset. They were included in the extraction if they generated new findings

## Results

### Current State of Literature

In recent years, there has been a significant increase in the number of publications addressing pregnancy in elite and sub-elite athletes. A particular rise has been observed over the last two decades, with 15 studies being published in the last five years alone (compared to nine studies between 1970 and 1999, and 22 studies between 2000 and 2019).

### Characteristics of Included Studies

Following a comprehensive search across multiple databases and citation tracking, a total of 5,236 records were identified (Fig. 1). After removing duplicates, 3,927 articles underwent title and abstract screening. Of these, 3,623 were excluded based on predefined criteria. Full texts of 302 articles were assessed for eligibility, resulting in the exclusion of 201 studies due to reasons such as absence of focus on pregnancy, lack of athlete populations (tier 3–5), policy-focused content, duplicate publication, or missing full-text availability (not even in the largest medical library in Germany, the Central Medical Library in Cologne).

Ultimately, 101 studies were included in the scoping review. Of these, 55 were categorized as review or overview articles (Appendix A3), while the remaining 46 were original research articles that underwent detailed data extraction. Compared to the last available scoping review [18], which included only 16 original studies, this review identified and extracted a total of 46 original studies for inclusion in the dataset. Further, compared to another review [19], which also incorporated all available literature (including reviews and mixed methods) and comprised 40 studies in total, this scoping review’s search strategy yielded 101 studies related to the topic of pregnancy and elite and sub-elite sport.

The extracted original studies comprised various designs: six were experimental studies, eight were cross-sectional studies, seven were observational cohort studies, four were case–control studies, nine were case reports or series, and 12 were qualitative studies. Six of the 46 studies were dataset duplicates, which were included in the data extraction due to different research questions or selection parameters.

### Mapping of Key Outcome Parameters

**Fig. 2:**
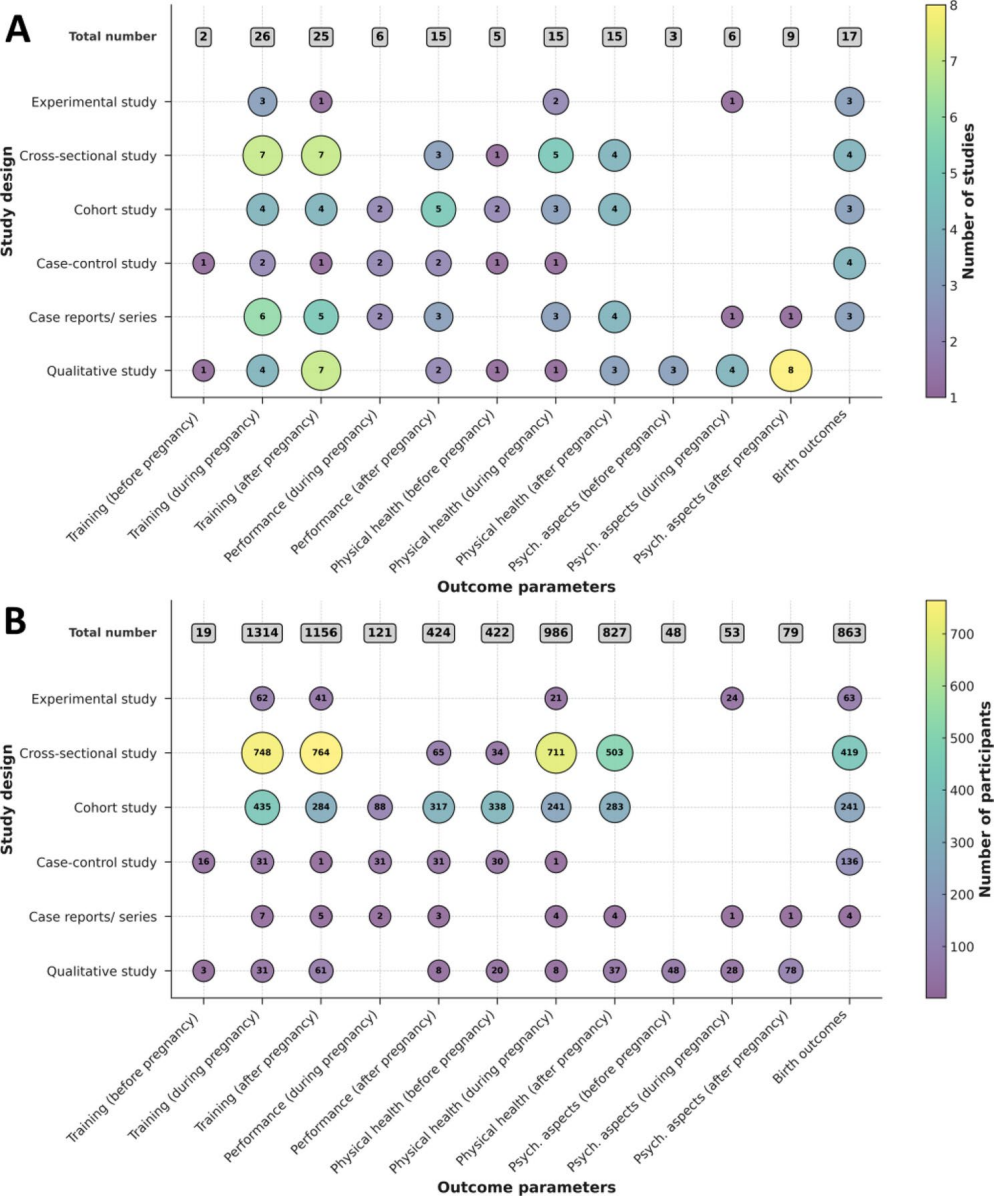
Frequency analysis. Key outcome parameters (training, performance, physical health, and psychological aspects) before, during, and after pregnancy, as well as birth outcomes are categorized by study design. Bubble size and numbers within the bubbles indicate either the number of studies (**A**) or the total sample size (**B**). The grey boxes above the figure show the total number of studies (**A**) and participants (**B**) for the different outcome parameters. The study designs on the y-axis are ordered by the strength of the evidence according to the 2011 Levels of Evidence from the Oxford Centre for Evidence-Based Medicine. Qualitative studies are shown separately at the bottom, as they do not fit into the hierarchy, but they provide valuable contextual and experiential insights.

Frequency analysis illustrates the distribution of outcome parameters such as training, performance, physical health, and psychological aspects before, during, and after pregnancy, as well as birth outcomes in relation to study design and sample size (Fig. 2). It is evident that there is a paucity of experimental studies (*n* = 5), and, among these, a particular absence of randomised controlled trials (*n* = 0). Furthermore, most cross-sectional and cohort studies are retrospective, which fundamentally reduces the strength of the overall evidence. A total of five experimental studies, eight cross-sectional studies, seven cohort studies, four case–control studies, nine case reports or series, and 11 qualitative studies were included in the frequency analysis. One experimental study and one qualitative study were excluded due to duplication.

The findings presented in this review must be interpreted with caution. The available evidence on elite and sub-elite athletes during pregnancy is still limited, often based on small or non-representative samples, and in several cases outdated. Training practices, medical guidance, and sport-specific recommendations for pregnant athletes have evolved markedly during this period. Consequently, the included studies reflect only a narrow segment of the actual experiences and conditions of pregnant athletes, which restricts the generalizability of the results.

### Before Pregnancy

#### Training

The available evidence is limited to a small number of studies with relatively small samples. These studies indicate that some athletes continue to train with comparatively high volumes and intensities until pregnancy is confirmed. In one study involving 34 Norwegian elite athletes, participants reported maintaining their usual training routines prior to pregnancy recognition [10]. Several recent studies indicate that women engage in 5 to 16 h of training per week prior to conception [10, 20, 21]. In specific disciplines such as running or judo, some athletes (24–57%) intentionally reduced training intensity or volume to support fertility or to align conception with planned career transitions [13, 22, 23].

#### Performance

Seven out of eight Olympic-level athletes typically planned their pregnancies to avoid disrupting their peak performance periods [13, 24]. Some athletes used the time during pregnancy to rest and recover from injuries [13]. In most cases, pregnancy is therefore planned very consciously [25].

#### Physical Health

Most athletes who engage in high-level training prior to pregnancy do not appear to experience negative effects on fertility or pregnancy outcomes [10, 24, 26]. One older study reported that 23% of endurance athletes experienced irregular menstrual cycles [27]. However, this finding should be interpreted cautiously given the publication date and limitations of the dataset. No more recent studies meeting the inclusion criteria reported menstrual irregularities in athletic populations before pregnancy. For elite athletes, planning for pregnancy can be particularly challenging, as they must balance reducing training to optimize fertility with maintaining competitive performance [23]. Overall, athletes are generally found to be healthier than non-athletic controls, displaying higher levels of education and lower rates of smoking and chronic health conditions [28].

### Psychological Aspects

The psychological burden of planning a pregnancy while pursuing an elite sport career can be significant. Many athletes reported being afraid of losing their team positions or sponsorships if they disclosed their pregnancies [23]. The social pressure to choose between motherhood and athletic ambition can lead to significant internal and external conflict [23, 25]. Peer support and sharing experiences with other athlete-mothers emerged as essential sources of reassurance and emotional resilience [21].

### During Pregnancy

#### Training

**Fig. 3:**
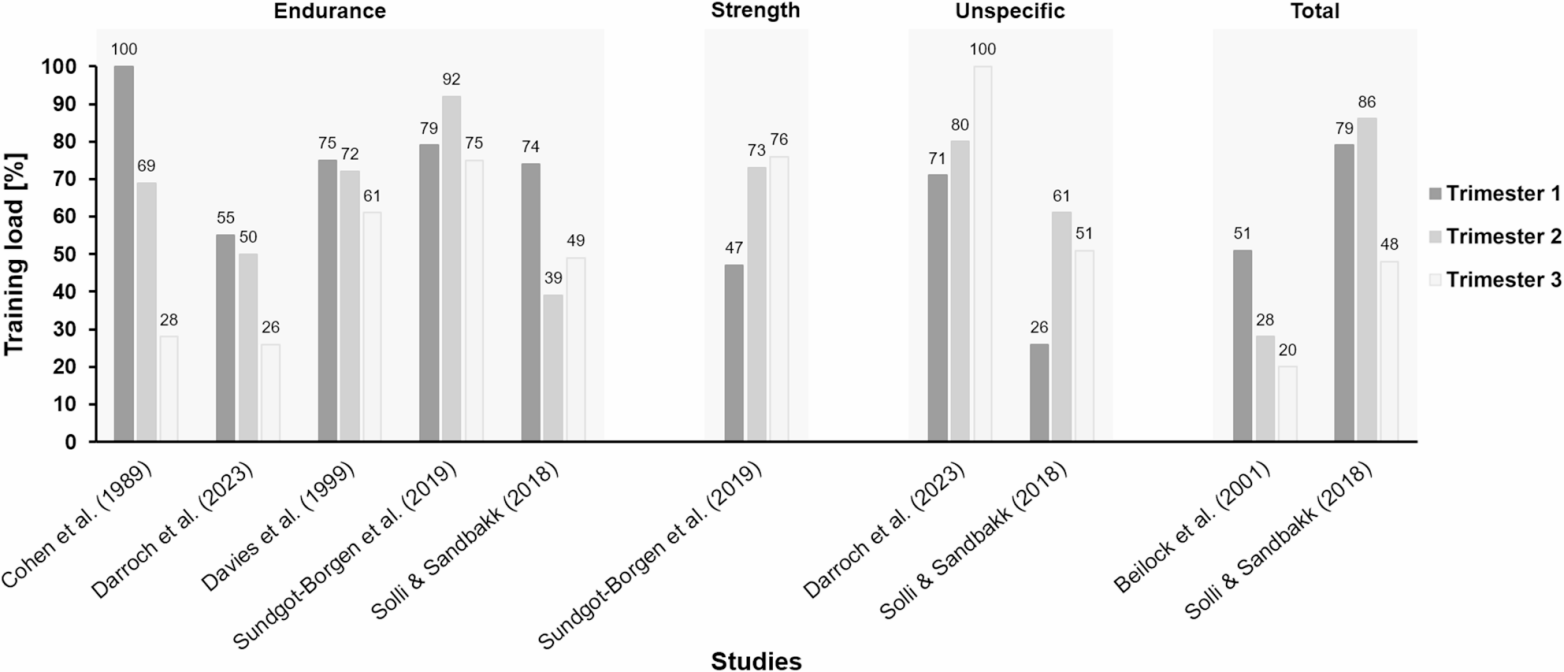
Training load distribution across pregnancy trimesters by training type. Percentage in training loads in different types of training (endurance training, strength training, unspecific or alternative training, and total training) in the 1st, 2nd and 3rd trimester as indicated in the respective studies. The percentages were either converted from the original data or read from the respective figures. Therefore, they should be interpreted accordingly. Cohen et al. (1989): n = 2, running (miles per week); Darroch et al. (2023): n = 42, running (kilometres per week); Davies et al. (1999): n = 1, running (kilometres per week) (twins); Sundgot-Borgen et al. (2019): n = 34, endurance training (minutes per week); Solli & Sandbakk (2018): n = 1, specific training (cross-country skiing, skating and classical) (hours per week); Sundgot-Borgen et al. (2019): n = 34, strength training (minutes per week); Darroch et al. (2023): n = 42, cross training (minutes per week); Solli & Sandbakk (2018): n = 1, unspecific training (running, walking, and cycling) (hours per week); Beilock et al. (2001): n = 26, cardio and strength training (FIT index); Solli & Sandbakk (2018): n = 1, overall training (hours per week).

Across the studies, the majority of high-level athletes continued to train during pregnancy. Training volume and intensity generally remained stable during the first trimester, but decreased gradually as the pregnancy progressed, particularly in the third trimester [10, 22, 29–32]. While sport-specific training usually decreased, non-specific training, such as cycling, walking, cross training, and strength training, increased (Fig. 3). Across endurance sports, training load typically declined from pre-pregnancy levels 0–30% in the first trimester to around 40–75% in the third trimester. Some studies reported a temporary increase in training load during the second trimester, which athletes attributed to feeling physically stronger and less symptomatic during this period [10, 32]. Reductions later in pregnancy were mainly linked to fatigue, mechanical discomfort, and decreased tolerance to impact-based activities [10, 33]. Training typically shifts towards lower-intensity, non-contact activities such as swimming, walking, and cycling, and some athletes compensated reduced running load through alternative, low-impact modalities. High-intensity or sport-specific activities, such as jumping and contact sports like judo or netball, were often stopped due to safety concerns or physical changes [21, 31, 32]. Given the limited available data, no robust comparison between weight-bearing and body-weight-supported endurance training could be made.

Several studies have investigated heart rate thresholds that are considered safe for training during pregnancy among elite and sub-elite athletes. In one older study published in 2001, 42% of participants reported using a conservative heart rate threshold of less than 140 beats per minute, whereas 39% preferred subjective measures such as perceived exertion rather than a fixed upper heart rate limit [29]. More detailed physiological assessments suggest that training within 50–75% of heart rate reserve is generally safe, with no adverse fetal outcomes reported [34]. One single study examined the effects of strenuous treadmill exercise at 60–90% maximum oxygen uptake (V̇O_2max_) during the second trimester (23–29 weeks of gestation) [35]. The study found that fetal heart rate remains within normal limits when the maternal heart rate stays below 90% of its maximum. However, exercise exceeding this threshold led to reduced arterial uterine blood flow and transient fetal bradycardia, which resolved post-exercise. This indicates that the maternal heart rate should remain below 90% of its maximum to minimize potential fetal compromise (35). In addition, another study showed that transient fetal heart rate decelerations and elevated umbilical and uterine Doppler indices occurred in five highly active pregnant women subsequent to strenuous treadmill exercise. However, no enduring impairment of fetal well-being was observed (36).

There is also evidence in older studies of longitudinal cardiovascular adaptations during pregnancy, including increased maternal heart rates during rest and submaximal exercise [37–39]. These findings of the 1990s suggest that pregnancy increases cardiovascular load even at submaximal intensities, highlighting the importance of personalized monitoring despite the lack of clearly defined heart rate thresholds. A considerable number of athletes (40–90%) have reported a preference for subjective metrics, such as perceived exertion, or “listening to the body” approaches. These strategies are frequently described as “go with the flow” approaches [21, 29, 31, 40].

Compared to the year prior to pregnancy, 11 out of 11 athletes do not reduce their weekly duration of low-intensity training during the first trimester compared to the year prior to pregnancy (452 ± 300 min per week) [31]. Most athletes stopped competing after the first trimester, citing physical changes and risk aversion as the main reasons [21, 27, 31]. Several studies indicate that elite athletes continued strength and core training during pregnancy, often modifying their training to accommodate physiological changes and mitigate injury risk. Training typically focused on the pelvic floor, abdominal and lower limb muscles, particularly in the later stages of pregnancy, to prevent dysfunction [31, 41, 42]. Athletes tended to choose low-risk activities such as swimming, cycling, and stabilization exercises while avoiding high-impact or abdominally stressful movements [21]. However, a lack of evidence-based guidance on strength training during pregnancy is frequently reported, leading many athletes to rely on peer networks or self-directed adaptations [10, 30]. More broadly, athletes consistently cite peer support or personal experience as their primary source of guidance, due to the ongoing absence of expert advice tailored specifically to high-level training [23, 30].

#### Performance

Some studies have examined the impact of pregnancy on athletic performance. Two case reports have observed increased cardiovascular responses, including elevated resting heart rate (12 bpm) and reduced V̇O_2max_ (30%) compared to postpartum, while submaximal performance capacity has remained largely stable (37,39). Another cohort study demonstrated that V̇O_2max_ remains stable during endurance testing in pregnancy, while resting oxygen uptake increases, maximum carbon dioxide production decreases and hyperventilation persists. A slight reduction in maximum heart rate (4 bpm) was also observed (43). A study comparing high- and medium-volume exercise groups during pregnancy found no significant differences in skinfold thickness, blood values, or perceived exertion. However, maternal resting heart rate increased during pregnancy, and only the high-volume group showed an improvement in oxygen consumption at maximal load. This finding suggests that, despite varying baseline capacities, there is comparable overall response to exercise (20).

Where competition behaviour was reported, 60% (18/30) of Finnish national-level endurance athletes continued to compete during pregnancy, on average until gestational week 13 (range 0–25 weeks) [27]. Similarly, Olympic athletes tended to suspend competitions near the end of the first or at the beginning of the second trimester [41]. Reasons for discontinuing competition included increasing symptoms, mechanical limitations and safety concerns, whereas those who continued typically reported feeling well and being closely supervised [27, 30, 31, 41]. While performance-focused training was generally avoided during this period, some athletes continued to engage in technical skill and strength training (30% increase [22]) [31, 42, 44].

#### Physical Health

The health effects of high-performance training during pregnancy are still being debated. The existing studies consistently report no adverse maternal or fetal health outcomes associated with moderate-intensity exercise [20, 27, 34]. However, this evidence base is limited and partially outdated, and the findings should therefore be interpreted cautiously. Although physiological adaptations such as an elevated maternal heart rate and slower core temperature recovery post-exercise have been documented, peak temperatures have remained within safe limits [45]. While moderate-intensity exercise is generally well tolerated, high-intensity training exceeding 90% of the maximum heart rate [35] and repeated sprint efforts [38] were identified as critical thresholds. Transient fetal bradycardia was observed in these instances. This evidence is partly based on a single case study [38] and must be understood within its historical context (1991), as both training methodologies and clinical recommendations have advanced substantially since that time.

While moderate-intensity exercise is generally well tolerated, high-intensity training exceeding 90% of the maximum heart rate [35] and repeated sprint efforts [38] were identified as critical thresholds. Transient fetal bradycardia was observed in these instances.

Serious complications are rare, with only single cases of mild pre-eclampsia, obstetric cholestasis, altered liver enzymes, and HELLP syndrome reported. None of these were conclusively linked to physical activity [35, 39, 46]. Other reported complications include general pregnancy disturbances (30%) [24], anemia (27%), and miscarriage or preterm birth (10.7%). However, no significant differences were found between athletes and controls [47]. One study published in 1997 reported that 23% of participants had previously experienced a spontaneous abortion, and five participants attributed this to intense training [27]. It is imperative to acknowledge that training practices and medical guidelines have undergone substantial modifications since the late 1990s.

Common symptoms during pregnancy include back pain, fatigue, nausea, dizziness, urinary incontinence, and musculoskeletal discomfort, particularly in the third trimester [21, 30, 38]. However, highly trained athletes reported similar or even fewer pregnancy-related complaints compared to the control groups [10, 44]. Although the overall injury incidence is low, individual cases of stress fractures, ankle sprains, and muscle strains have been documented [10]. Notably, a study revealed that 98.2% of athletes in one cohort reported no injuries during pregnancy [41].

The average gestational weight gain among elite and sub-elite athletes is between 9 and 14 kg. Those engaged in high-level competition are more likely to exceed the Institute of Medicine’s (IOM) recommendations slightly (13.7–16.1 kg) [41, 48]. The increased likelihood of competitive exercisers exceeding the IOM recommendations for gestational weight gain may partially be explained by a shift towards a positive energy balance, as many women with high pre-pregnancy activity levels substantially reduce their training volume during pregnancy without a corresponding reduction in energy intake [48]. In addition, competitive athletes often have a lower pre-pregnancy fat mass, which may predispose them to larger gains in fat tissue once training loads are reduced [48].

Remarkably, the majority of studied athletes (96%) reported an overall uncomplicated pregnancy experience with few or no significant symptoms [41].

#### Psychological Aspects

Psychologically, pregnancy is a time of heightened uncertainty and emotional ambivalence for elite athletes. Many reported increased anxiety due to a lack of evidence-based guidance on safe training, coupled with fears that continued physical activity might affect fertility or the health of the fetus [23, 30, 49]. The social and institutional pressure to choose between elite sport and motherhood intensifies this emotional strain further, contributing to conflicts of identity and concerns about career security [23, 25, 50]. Qualitative studies indicate that, in some contexts, pregnant elite athletes face loss of sponsorship and sport funding, and may risk losing a substantial part of their income in the absence of maternity policies. This financial insecurity can create pressure to maintain training or to return to competition shortly after childbirth [7, 23]. Athletes often struggle with changes in body image and self-perception, as well as fears of a decline of performance [23, 49]. However, studies also highlight the potential benefits of psychopedagogical interventions, such as relaxation training and counseling, which can significantly reduce anxiety and depressive symptoms during pregnancy [51].

Despite these psychological challenges, some athletes reported positive emotional engagement with their changing bodies, describing pregnancy as a time of physical and mental renewal [13]. Notably, peer support and shared experiences with other athlete mothers emerge as crucial sources of reassurance, motivation, and resilience throughout the pregnancy journey [21].

### After Pregnancy

#### Training

The time it takes to return to training after childbirth varies from person to person, but it often happens earlier than the return to recreational sports in the general population. Many athletes resumed light activities, such as pelvic floor exercises, walking, or functional workouts, within the first six weeks after childbirth [10, 21, 42]. Across the available cohorts, most elite athletes resumed training within the first 6–12 weeks postpartum. In a sample of Norwegian elite athletes, 71% had returned to sport or exercise by 0–6 weeks and a further 24% by 7–12 weeks postpartum (i.e. 95% within 3 months) [10]. Elite runners in another cohort resumed running after 6 ± 6 weeks and reached ~ 80% of their prepregnancy training load by 14 ± 11 weeks postpartum [22], and in a smaller Swiss sample 70% of athletes had reinitiated low-intensity endurance training within 1 month after vaginal delivery, with most high-intensity sessions resumed between 6 and 8 weeks postpartum [31]. The pre-pregnancy training volume was usually achieved within 3–9 months [24, 31, 41]. The time taken to return to training after a cesarean birth varies [31, 47]. In one of the largest athlete cohorts available with 328 former female competitive athletes, those who had undergone a cesarean section returned to competition after an average of 10.0 ± 13.3 months, which was not significantly different from athletes with vaginal births (10.6 ± 21.8 months) [47]. Studies also report coordination difficulties due to sleep deprivation and breastfeeding, which impacts training [22, 52]. Darroch et al. (2023) observed that those who maintained higher training volumes in the first and second trimesters had better postnatal performance outcomes [22].

#### Performance

The postpartum period can be accompanied by the following physiological changes: a decrease in maternal heart rate, an increase in blood lactate concentrations, an early peak in oxygen uptake, and a slight reduction in vital capacity [37]. In the only study reporting longitudinal values, resting heart rate rose from 56 to 68 bpm during pregnancy and decreased to 48 bpm by 6 weeks postpartum, indicating a return toward (or below) baseline [37].

Many athletes successfully returned to their peak athletic performance, albeit at different speeds. Studies have shown that athletes can regain their pre-pregnancy performance levels [24, 40, 41] or even achieve personal bests [22, 29, 38, 53]. One study found that 11% of athletes improved, 61% returned to the same level of performance, and 28% did not return to their previous level of performance after pregnancy [27]. In another study, 44% of athletes indicated that they had returned to the same level of performance, 15% reported an improvement in performance, while 26% exhibited a deterioration, and 15% were unsure [10]. The rate of recovery is significantly influenced by several factors, including the duration of breastfeeding, the volume of training during pregnancy and the postpartum period, and individual characteristics [22]. Additionally, the ability to return to sport and improve performance primarily depends on the age at which the athletes become pregnant, relative to their respective peak performance ages [53]. Postpartum sprint intervals or targeted endurance training have been shown to rapidly restore cardiovascular markers [54, 55].

#### Physical Health

Typical postpartum health issues affect the musculoskeletal system, causing problems such as pelvic floor weakness, rectus diastasis, and stress fractures, as well as back pain and incontinence [13, 47, 56]. Despite these challenges, many athletes reported a stable recovery and return to normal physical performance within a year [10, 57]. Breastfeeding, although logistically demanding, is often continued alongside training; however, potential impacts on performance may arise from sleep disruption or challenges related to milk supply. At this stage, it is crucial to ensure that athletes are drinking enough fluids [12, 52]. Only a few studies addressed nutritional aspects during pregnancy or postpartum. In a qualitative study of elite distance runners, athletes expressed concerns regarding breast milk composition, the potential effects of lactic acid accumulation after high-intensity exercise, and the difficulty of meeting increased energy demands while breastfeeding and maintaining training levels [52]. Similarly, a recent qualitative study involving UK elite athletes reported a general lack of tailored postpartum nutrition guidance, with many athletes feeling insufficiently supported in managing energy intake, supplementation practices, and weight regulation during their return to sport [12].

#### Psychological Health

The psychological outcomes in the studies are mixed: female athletes reported newly gained strength and determination as a result of motherhood. However, they also report feelings of insecurity, excessive demands, and depressive symptoms, particularly due to social expectations and the tension between career and motherhood [12, 56, 58]. Motherhood was reported to be perceived as a transformative experience [59]. Athletes reported improved life balance, increased motivation, and a redefined athletic identity [40, 58]. Emotional struggles include feelings of guilt, pressure, and difficulties to return to sport, loss of squad status and difficulty reconciling changes to the body with athletic goals [12, 50, 56]. Emotional and mental support from the immediate environment was specified as a key resource [50]. The identity of being both “athlete” and “mother” was often redefined and served as a source of motivation and inspiration [58]. However, a lack of professional psychosocial support remains a significant issue [59, 60].

### Birth Outcomes

**Fig. 4:**
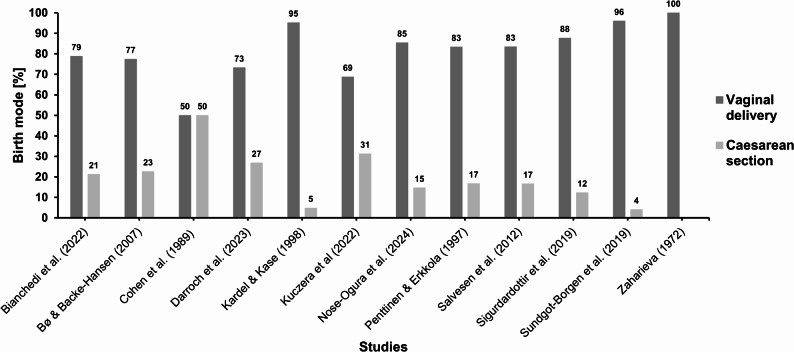
Birth mode. Frequency distribution of two birth modes among elite and sub-elite athletes in selected studies: vaginal births (including induced labor and assisted deliveries) and cesarean sections (including elective and spontaneous procedures).

The birth outcomes of highly trained athletes appear to be largely comparable to, or even slightly better than, those of the general population. Several studies reported high rates of spontaneous vaginal delivery (~ 70%) (Fig. 4) and healthy Apgar scores after five minutes (> 7), with no differences observed compared to controls (70–75%) [34, 41, 47]. Cesarean section rates vary, but are not elevated in most elite and sub-elite cohorts compared to controls [26, 41, 61].

Importantly, no consistent negative effects of intense or sustained training on labor or neonatal outcomes have been demonstrated. Studies comparing elite and sub-elite athletes to non-athletes showed no significant differences in birth mode, duration of labor, need for interventions, complications or neonatal health metrics [24, 27, 61, 62]. However, some data suggest an increased likelihood of prolonged labor among highly trained women, potentially due to stronger pelvic musculature [24].

**Fig. 5:**
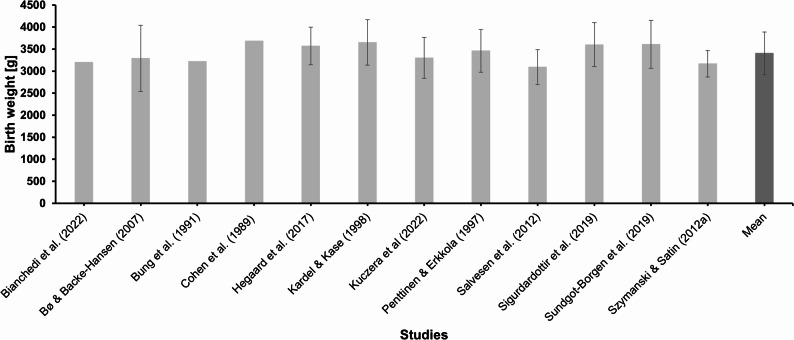
Birth weight. Mean values (light grey bars) and, if available, the respective standard deviations of birth weights of babies from elite and sub-elite athletes in selected studies. The mean value of all studies is represented by the dark grey bar.

Birth weight generally falls within normal ranges, with an average value of 3.403 ± 487 g (Fig. 5). However, contradictory findings have been reported in the literature. Some studies reported slightly higher weights in conjunction with increased maternal exercise intensity [29], while others reported lower weights in endurance athletes with high training volume [35]. Perineal injuries and interventions (e.g., episiotomies) were reported in line with general trends and were not consistently correlated with athletic status [26, 61].

Overall, the available evidence supports the idea that participation in elite-level sports during pregnancy is not associated with adverse birth outcomes, provided that training intensity and volume is appropriately moderated and medical supervision is in place.

## Discussion

This review provides a unique, comprehensive, and up-to-date overview of the current state of research on pregnancy and elite sports. By systematically searching 10 different databases, a more extensive array of original articles has been included than in any previous review on the topic. Furthermore, it identifies and discusses existing research gaps with the aim of fostering systematic development in original experimental research studies.

This work reveals the growing prominence of pregnancy in elite and sub-elite sports, while also highlighting significant gaps that hinder the development of concrete, evidence-based guidelines. Notably, much of the current literature is predicated on a limited number of original studies, emcompassing limited sample sizes, single-case reports, or retrospective designs. This severely restricts the ability to generalize, particularly with regard to training load, intensity thresholds during pregnancy, and the timing of the postpartum return to sport as well as birth outcomes, and fertility issues.

### Training Load

The ongoing reliance on subjective metrics, including perceived exertion and self-monitoring, underscores a notable absence of standardized physiological benchmarks [21, 29, 31, 40]. Recommendations on maternal heart rate during pregnancy, for example, are predominantly derived from single experimental studies [34, 35] and cannot yet serve as robust guidelines. Similarly, the hypothesis that training below 90% of maximum heart rate [35] or within 50–75% of heart rate reserve [34] is “safe” is not supported by high-level evidence. This hypothesis requires replication and confirmation through larger controlled trials. The absence of standardized, sport-specific recommendations means that athletes depend significantly on self-monitoring and peer advice [21, 23]. While this strategy emphasizes physical autonomy, it can also expose athletes to the risks of overtraining, injury, and inadequate postpartum recovery. In our view, the assertion that moderate exercise intensity can be gauged by the ability to still speak during exercise (the “talk test”) or by means of perceived exertion scales [63] is inadequate for evidence-based recommendations concerning training management.

With respect to the management of training loads, the data indicates that high-level athletes adjust their training volume, intensity, and type during pregnancy. However, the optimal “safe” ranges remain unresolved. Furthermore, a systematic comparison with general recommendations for recreationally active pregnant women has not been undertaken [31]. The systematic documentation of weekly training loads, as referenced in studies [32, 42, 46, 55], is needed. The development of single sport-specific recommendations on training adjustments during pregnancy appears to be an unrealistic objective at this time. Consequently, further research is necessary into specific sports groups, including endurance sports, team sports, high-impact sports, and explosive strength sports.

Ideally, phase-specific documentation of training and health data should be organized according to gestational age in weeks, or at least in trimesters, to help move beyond inconsistent descriptive data and derive general recommendations. Recommendations regarding resistance training during pregnancy are neither uniform nor consistently evidence-based. Thus, the question remains as to how muscle strength and muscle tone, particularly that of the pelvic floor, influence the course of labor and delivery [64]. For instance, it is unclear whether strengthening the already strong pelvic floor of elite athletes would have a positive or negative impact on the progression of childbirth.

### Return to Sport

Postpartum return to sport patterns are highly individualized. While many athletes successfully return to or surpass pre-pregnancy levels of training loads and performance [22, 41, 53], this process is influenced by factors, such as breastfeeding, sleep deprivation, injury history, and psychosocial support [52, 56]. However, the current evidence base is largely comprised of self-reported retrospective outcomes, which introduces the potential for bias that could mask underlying vulnerabilities, particularly with regard to pelvic floor health or injury recurrence [13, 47]. Interestingly, athletes often report fewer common pregnancy-related issues than recreationally active women, potentially due to superior baseline fitness and body awareness [10]. Nevertheless, clear medical guidance on sport-type-specific risks and considerations is lacking and urgently needed, particularly in disciplines where body mass plays a crucial role (e.g., aesthetic or weight-class sports) or where strong abdominal muscles are essential, especially with regard to injury prevention and pelvic floor health.

### Psychological Dimensions

In terms of psychological aspects, motherhood can be a source of both empowerment and significant stress [58–60]. Identity conflicts, social pressures, and sponsorship concerns can exacerbate these challenges [12, 23]. Data suggest that successful return to sport depends on physical readiness as well as psychosocial, logistical, and financial support systems. Having individualized recovery plans that set out the competencies to be achieved during the postpartum period, alongside a structured reintegration into competition, could reduce risks, and support sustainable performance improvements. While there is some evidence to suggest psychological benefits, such as enhanced resilience [40], the formal evaluation of mental health interventions using standardized tools, such as validated questionnaires, remains rare [51].

### Birth Outcomes

Finally, the birth outcomes of highly trained athletes seem to be largely similar to those of the general population [26, 41, 61]. However, it should be noted that these findings are based on small, heterogeneous samples, so they should be interpreted with caution. Observations regarding prolonged labor or slight deviations in neonatal weight suggest potential physiological adaptations [24, 35]. Nevertheless, further systematic research specific to different sports is essential before definitive conclusions can be drawn.

### Fertility

There are inconclusive findings on how high-performance sport affects fertility. Athletes can generally maintain high training loads without apparent reproductive impairment when energy availability is sufficient [10], whereas the Relative Energy Deficiency Syndrome in Sports (REDs) is associated with menstrual and ovulatory dysfunction independent of training volume [65]. Importantly, most included studies examined only athletes who achieved pregnancy, leaving potential fertility problems and early pregnancy losses underreported. Given the likelihood of unrecorded cases, partly due to social stigma and a paucity of open discussion [66, 67], and limited systematic data, reproductive health outcomes in elite athletes remain insufficiently understood and warrant more rigorous investigation.

#### Research Gaps and Future Directions

In light of these gained insights, future research should prioritize high-quality experimental and prospective studies involving larger and more diverse cohorts of athletes. Interventions combining individualized training prescriptions, standardized physiological monitoring, and comprehensive psychosocial support should be systematically developed and evaluated. Furthermore, long-term follow-up is necessary to assess sustained health and performance trajectories, as well as immediate postpartum outcomes.

The present review emphasizes the necessity of systematic, in-depth descriptive documentation, and the advancement of more mechanistic and interventional research. Closing these evidence gaps is essential to developing meaningful, sports group-specific guidelines that go beyond anecdotal strategies and provide genuine support for athletes and mothers at the highest level.

**Fig. 6:**
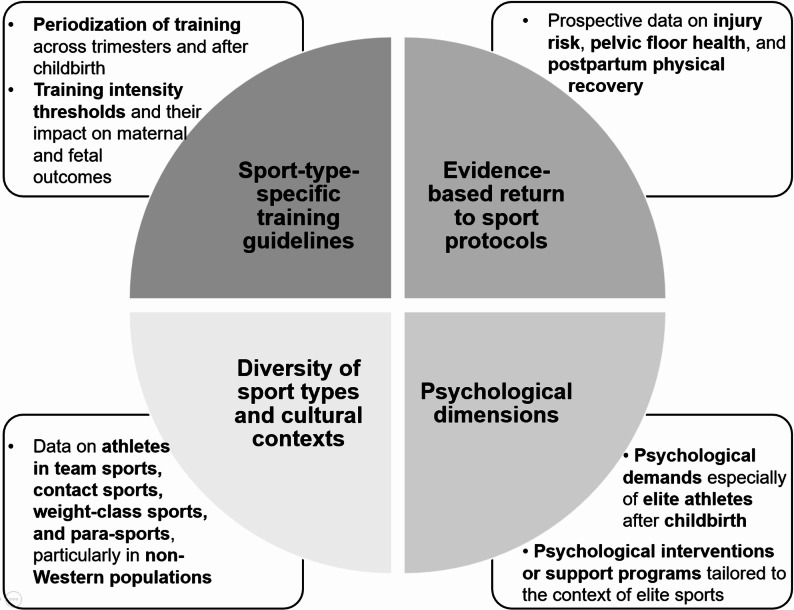
Key research gaps identified in this scoping review on pregnancy in elite sports

Notwithstanding the mounting involvement of women in high-performance sports, the available evidence remains fragmented (Fig. 6). The majority of studies utilize retrospective designs, small sample sizes, and self-reported data. Notably, there is a lack of prospective, sports group-specific studies examining the physiological, psychological, and performance-related changes across the entire peri-pregnancy timeline. A paucity of studies exists that provide structured, evidence-based return to sport protocols, and even fewer address the long-term career trajectories of athlete mothers. Furthermore, psychological dimensions such as identity negotiation, postpartum mental health, and societal expectations are underrepresented in quantitative research. The current literature also favors endurance sports in Western contexts and offers limited insights into team sports, weight-class sports, or para-sport disciplines. Addressing these disparities is imperative for the development of effective, personalized support systems and policies that reflect the realities of elite sport and motherhood.

Longitudinal, prospective studies with larger sample sizes are urgently needed to generate robust data that can inform policy, medical practice, and coaching strategies. The extant body of research is predominantly composed of retrospective, qualitative, or single-case studies. However, the dearth of studies employing longitudinal, controlled, or interventional designs, compromises the extent to which robust conclusions can be drawn. Additionally, the absence of standardized outcome measures across studies complicates the synthesis and comparison of findings, impeding the establishment of clear conclusions.

## Conclusion

This scoping review underscores the intricate interplay of physiological, psychological, and social factors that influence the experiences of elite female athletes during pregnancy and the postpartum period. Despite the increasing recognition of the advantages of continuing to train during pregnancy, elite athletes are still not receiving the evidence-based, sport group-specific guidelines they need to address their unique requirements and performance objectives. The extant literature underscores the remarkable physical adaptability of athlete-mothers and their promising return to performance trajectories. However, these positive outcomes are frequently attained in the absence of standardized medical and coaching frameworks, thereby exposing athletes to potential risks, particularly relating to injury and inadequate postpartum recovery.

Furthermore, the transition to motherhood constitutes a substantial psychological and social transformation. While this phenomenon may be empowering for some, it concomitantly engenders significant emotional challenges. The dual identity of athlete and mother has been demonstrated to be a source of resilience and renewed motivation. However, there is a clear need for targeted mental health support and integrated psychosocial interventions.

This review uncovers significant gaps in the current evidence base, particularly with regard to prospective, sport group-specific research across diverse athletic disciplines and cultural contexts. Importantly, although no consistent evidence of adverse pregnancy outcomes associated with high training loads has been reported, the available data are too limited, heterogeneous, and heavily focused on endurance sports to support firm conclusions, particularly for resistance-based or high-intensity disciplines. These studies are necessary to inform the development of tailored training adaptations, return to sport protocols, and multidisciplinary support strategies. Addressing these disparities is imperative for developing comprehensive, personalized guidelines that facilitate the safe and sustainable pursuit of athletic excellence by elite athletes while ensuring the optimal conditions for motherhood.

In order to accomplish the aforementioned research objectives, it is imperative to establish an international collaborative research network. This network will assure that sample sizes are sufficiently large and diverse across sports, regions, and performance levels.

## Supplementary Information

Below is the link to the electronic supplementary material.

Supplementary Material 1

## Data Availability

All the data analyzed during this scoping review are included in the published article, in the tables that summarize the included studies. The corresponding author can provide reasons for the exclusion of studies.

## Abbreviation

V̇O_2max_: Maximum oxygen uptake
