# Supplementary material for: Elite and Sub-elite Athletes and Pregnancy: Training, Performance, Health and Psychological Aspects Across the Pre-, Peri-, and Postnatal Stages: A Scoping Review
Source: Sports Med Open. 2026 Mar 9;12:25. doi: 10.1186/s40798-026-01000-5 (PMC12972377; doi:10.1186/s40798-026-01000-5)
Supplement: Supplementary file 1 — Supplementary Material 1 [file 40798_2026_1000_MOESM1_ESM.pdf]

Journal: Sports Medicine - Open

Elite and Sub-elite Athletes and Pregnancy: Training, Performance, Health and Psychological Aspects the Across Pre-, Peri-, and Postnatal Stages - A Scoping Review

Jana Nolte[1, *], Isabell Thal[1], Emily B ˇ the[1], Susanne Weber[2], Petra Platen[1], & Kirsten Legerlotz[3]

[1] Department of Sports Medicine and Sports Nutrition, Ruhr University Bochum, Bochum, Germany

[2] Practice for Gynecology and Obstetrics, specialized in Sports and Exercise Gynecology, Heidelberg/Schriesheim

[3] Department of Movement and Training Sciences, University of Wuppertal, Wuppertal, Germany

* Correspondence:

Jana Nolte

jana.nolte@rub.de

Appendix

A1 Population description:

This scoping review focused on elite female athletes, as defined by McKay et al. (2019). This classification system categorizes athletes into tiers 0-5 based on training volume, competitive level, and athletic performance. Athletes were classified as tiers 3ό5 (i.e., highly trained to world-class athletes), or were included if they reported a minimum training volume of eight hours per week, indicative of a high-performance regimen. Where studies did not explicitly assign athletes to a tier, they were categorized based on additional indicators, such as reported training frequency, competitive achievements or international ranking.

Table A1: The classification system used (adapted from McMay et al., 2022)

Tier	Description	Characteristics of an athlete
Tier 1	Recreationally active	Engages in physical activity or general fitness, no competition
Tier 2	Trained	Trains regularly, may compete at local level, not performance-oriented
Tier 3	Highly Trained/National Level	Conducts regular, structured training, competes at national competitions and/or in the highest regional (e.g., state or provincial) leagues that feed into national level competition. Typically engaging in structured, periodized training and their performance levels are generally within 20% of the best performances worldwide.
Tier 4	Elite/International Level	Conducts systematic, high-volume training, competes at national or international level
Tier 5	World-class	Trains with maximal specialization and focuses on performance, competes at the highest international level, such as the Olympics or World Championships

Where studies did not report an explicit tier classification or detailed competitive level, athletes were ranked based on individual training hours, performance benchmarks (e.g. race times and

rankings), and contextual information regarding training infrastructure and support systems. Where transparent information was lacking, the classification was made conservatively. These classifications were marked with an asterisk (*) to denote an inferred assignment. Mixed groups were defined as study samples containing at least one elite (tiers 3ó5) athlete, as well as other highly active women who did not meet the strictest criteria for being considered elite. Such groups were identified either through explicit mention (î competitiveï, î professionalï, or î semi-professionalï athletes) or inferred based on reported training volumes and competitive backgrounds. These groups were included in the analysis, but labeled as î mixed groupï to distinguish them from samples containing elite athletes only. This rigorous classification approach aimed to ensure that only data reflecting the specific experiences and challenges of elite-level athleticism during pregnancy and the postpartum period were synthesized, while maintaining sufficient inclusivity to reflect the diversity of contexts in high-performance sports.

A2 Search Terms

(pregnant OR pregnancy OR prepartum OR antepartum OR peripartum OR prenatal OR antenatal OR perinatal OR postnatal OR obstetric OR birth OR fetus OR preconception)

AND

(athlete OR elite OR olympic OR competitive sport OR runner OR marathon OR triathlon OR swimmer OR cyclist OR endurance training OR strength training OR high intensity interval training OR team sport OR combat sport)

Filters Applied

- Population: Humans

- Language: English, German, French, Spanish

A2 Databases, date of search, and number of hits

PubMed including MEDLINE, 08.01.2025

Search	Query	Hits
#1	(pregnant OR pregnancy OR prepartum OR antepartum OR peripartum OR prenatal OR antenatal OR perinatal OR postnatal OR obstetric OR birth OR fetus OR preconception)	1,907,041
#2	(athlete OR elite OR olympic OR competitive sport OR runner OR marathon OR triathlon OR swimmer OR cyclist OR endurance training OR strength training OR high intensity interval training OR team sport OR combat sport)	508,719
#3	#1 AND #2	9,738
#4	#3 [Title/Abstract]	726
#5	#4 Filters: Humans, English, French, German, Spanish	441

Cochrane, 08.01.2025

Search	Query	Hits
#1	(pregnant OR pregnancy OR prepartum OR antepartum OR peripartum OR prenatal OR antenatal OR perinatal OR postnatal OR obstetric OR birth OR fetus OR preconception)	125,611
#2	(athlete OR elite OR olympic OR competitive sport OR runner OR marathon OR triathlon OR swimmer OR cyclist OR endurance training OR strength training OR high intensity interval training OR team sport OR combat sport)	39,423
#3	#1 AND #2	1,497
#4	#3 :ti;ab;kw*	507

*ti: Title; ab: Abstract; kw: Keywords

Web of Science, 08.01.2025

Search	Query	Hits
#1	(pregnant OR pregnancy OR prepartum OR antepartum OR peripartum OR prenatal OR antenatal OR perinatal OR postnatal OR obstetric OR birth OR fetus OR preconception)	1,378,286
#2	(athlete OR elite OR olympic OR competitive sport OR runner OR marathon OR triathlon OR swimmer OR cyclist OR endurance training OR strength training OR high intensity interval training OR team sport OR combat sport)	409,697
#3	#1 AND #2	3,743
#4	#3 (Abstract)	1,873
#5	#4 and English or Spanish or German or French (Languages)	1,808

APA PsycNet (PsycArticles + PsycBooks), 08.01.2025

Search	Query	Hits
#1	(pregnant OR pregnancy OR prepartum OR antepartum OR peripartum OR prenatal OR antenatal OR perinatal OR postnatal OR obstetric OR birth OR fetus OR preconception)	38.823
#2	(athlete OR elite OR olympic OR competitive sport OR runner OR marathon OR triathlon OR swimmer OR cyclist OR endurance training OR strength training OR high intensity interval training OR team sport OR combat sport)	1.936
#3	#1 AND #2	154
#4	#3 Abstract:	4
#5	#4 Filters: Human	4

Scopus, 08.01.2025

Search	Query	Hits
#1	(pregnant OR pregnancy OR prepartum OR antepartum OR peripartum OR prenatal OR antenatal OR perinatal OR postnatal OR obstetric OR birth OR fetus OR preconception)	4,368,258
#2	(athlete OR elite OR olympic OR competitive sport OR runner OR marathon OR triathlon OR swimmer OR cyclist OR endurance training OR strength training OR high intensity interval training OR team sport OR combat sport)	24,206
#3	#1 AND #2	1,624
#4	#3 TITLE-ABS-KEY	3
#5	#4 Filters: Human	3

SPONET, 13.01.2025, Search in English

Search	Query	Hits
#1	(pregnant OR pregnancy OR prepartum OR antepartum OR peripartum OR prenatal OR antenatal OR perinatal OR postnatal OR obstetric OR birth OR fetus OR preconception)	524
#2	(athlete OR elite OR olympic OR competitive sport OR runner OR marathon OR triathlon OR swimmer OR cyclist OR endurance training OR strength training OR high intensity interval training OR team sport OR combat sport)	480
#3	#1 AND #2	6

SPONET, 13.01.2025, Search in German

Search	Query	Hits
#1	(schwanger OR Schwangerschaft OR prÊpartal OR antepartal OR peripartal OR prÊnatal OR antenatal OR perinatal OR postnatal OR Geburtshilfe OR Geburt OR F˘tus OR EmpfÊngnisvorbereitung)	154
#2	(Athletin OR Elite OR Olympia OR Leistungssport OR LÊuferin OR Marathon OR Triathlon OR Schwimmerin OR Radfahrerin OR Ausdauertraining OR Krafttraining OR hochintensives Intervalltraining OR Mannschaftssport OR Kampfsport)	47,458
#3	#1 AND #2	49

SURF, 13.01.2025, Search in English

Search	Query	Hits
#1	(pregnant OR pregnancy OR prepartum OR antepartum OR peripartum OR prenatal OR antenatal OR perinatal OR postnatal OR obstetric OR birth OR fetus OR preconception)	1,548
#2	(athlete OR elite OR olympic OR competitive sport OR runner OR marathon OR triathlon OR swimmer OR cyclist OR endurance training OR strength training OR high intensity interval training OR team sport OR combat sport)	2,296
#3	#1 AND #2	7

SURF, 13.01.2025, Search in German

Search	Query	Hits
#1	(schwanger OR Schwangerschaft OR prÊpartal OR antepartal OR peripartal OR prÊnatal OR antenatal OR perinatal OR postnatal OR Geburtshilfe OR Geburt OR F˘tus OR EmpfÊngnisvorbereitung)	1,177
#2	(Athletin OR Elite OR Olympia OR Leistungssport OR LÊuferin OR Marathon OR Triathlon OR Schwimmerin OR Radfahrerin OR Ausdauertraining OR Krafttraining OR hochintensives Intervalltraining OR Mannschaftssport OR Kampfsport)	76,238
#3	#1 AND #2	249

PsycINFO, 13.01.2025

Search	Query	Hits
#1	(pregnant OR pregnancy OR prepartum OR antepartum OR peripartum OR prenatal OR antenatal OR perinatal OR postnatal OR obstetric OR birth OR fetus OR preconception)	184,049
#2	(athlete OR elite OR olympic OR competitive sport OR runner OR marathon OR triathlon OR swimmer OR cyclist OR endurance training OR strength training OR high intensity interval training OR team sport OR combat sport)	59,080
#3	#1 AND #2	704
#4	AB: (#3)	379
#5	#4 Filters: Human; English or Spanish or German or French	345

Ovid EMBASE, 29.01.2025

Search	Query	Hits
#1	(pregnant OR pregnancy OR prepartum OR antepartum OR peripartum OR prenatal OR antenatal OR perinatal OR postnatal OR obstetric OR birth OR fetus OR preconception)	
#2	(athlete OR elite OR olympic OR competitive sport OR runner OR marathon OR triathlon OR swimmer OR cyclist OR endurance training OR strength training OR high intensity interval training OR team sport OR combat sport)	
#3	#1 AND #2	1814

A3 List of overview articles

Our search strategy identified 55 publications that were considered to be secondary literature. These included reviews, meta-analyses, clinical guidelines, book chapters, digests, infographics, media analysis and commentaries.

1. Afifi T, Barrack MT, Casey E, Huddle M, Kliethermes SA, Kraus E, u. a. Infographic. Head to toe considerations for the postpartum endurance athlete. Br J Sports Med. 2024;58(11):63062.
2. Araujo D. Expecting Questions About Exercise and Pregnancy? The Physician and Sportsmedicine. 1997;25(4):84693.
3. Baldwin K. Run like a mother: running, race, and the shaping of motherhood under Covid-19. Feminist Media Studies. 2023;23(6):2546661.
4. Bilal A, Ahmed Z, Mohsin M, Fazal A, Khan S, Saeed S, u. a. Guidelines for Physical Activity and Exercise for Women with Hyperglycemia in Pregnancy. Journal of Diabetology. 2021;12(Suppl 1):S9267.
5. Bischoff LL. Frauen im Spitzensport: Forschungsl˝cken und methodische Herausforderungen. Zeitschrift f˝r Sportpsychologie. 2022;29(263):1076107.
6. Bland JH, Casey MJ. WOMEN IN WINTER SPORTS. In: Winter Sports Medicine. Philadelphia (Penn.) F.A. Davis; 1990. S. 42655.
7. B˚ K, Artal R, Barakat R, Brown W, Davies GAL, Dooley M, u. a. Exercise and pregnancy in recreational and elite athletes: 2016 evidence summary from the IOC expert group meeting, Lausanne. Part 1ò exercise in women planning pregnancy and those who are pregnant. Br J Sports Med. 2016a;50(10):571689.
8. B˚ K, Artal R, Barakat R, Brown WJ, Davies GAL, Dooley M, u. a. Exercise and pregnancy in recreational and elite athletes: 2016/17 evidence summary from the IOC Expert Group Meeting, Lausanne. Part 3ò exercise in the postpartum period. Br J Sports Med. 2017;51(21):1516625.
9. B˚ K, Artal R, Barakat R, Brown WJ, Davies GAL, Dooley M, u. a. Exercise and pregnancy in recreational and elite athletes: 2016/17 evidence summary from the IOC expert group meeting, Lausanne. Part 4ò Recommendations for future research. Br J Sports Med. 2017;51(24):172466.
10. B˚ K, Artal R, Barakat R, Brown WJ, Davies GAL, Dooley M, u. a. Exercise and pregnancy in recreational and elite athletes: 2016/2017 evidence summary from the IOC expert group

meeting, Lausanne. Part 5. Recommendations for health professionals and active women. Br J Sports Med. 2018;52(17):108065.

11. Borowski LE, Barchi EI, Han JS, Friedman DA, Carter CW. Musculoskeletal Considerations for Exercise and Sport: Before, During, and After Pregnancy. J Am Acad Orthop Surg 2021: https://journals.lww.com/10.5435/JAAOS-D-21-00044
12. Carmichael RD. Considerations for the Pregnant Endurance Athlete. Strength & Conditioning Journal. 2021;43(6):35641.
13. Caron J, Crozier A, Ede A, Hoffman M, Lee S, Leisterer S, u. a. Digest. Gammage K, Herausgeber. Journal of Sport & Exercise Psychology. 2023;45(4):23468.
14. Commentaries on Viewpoint: Are there valid concerns for completing a marathon at 39 weeks of pregnancy? Journal of Applied Physiology. 1. Oktober 2012;113(7):116661166.
15. Constantini NW, Warren MP. Special problems of the female athlete. BailliÍreìs Clinical Rheumatology. 1994;8(1):1996219.
16. Deering RE, Christopher SM, Heiderscheit BC. From Childbirth to the Starting Blocks: Are We Providing the Best Care to Our Postpartum Athletes? Journal of Orthopaedic & Sports Physical Therapy. 2020;50(6):28164.
17. Donnelly G, Coltman C, Dane K, Elliott□Sale K, Hayman M, McCarthy□Ryan M, u. a. Prioritise safety, optimise success! Return to rugby postpartum. European Journal of Sport Science. 2024;24(12):1701618.
18. Erdener U, Budgett R. Exercise and pregnancy: focus on advice for the competitive and elite athlete. Br J Sports Med. 2016;50(10):5676567.
19. Fieseler CM. Special considerations for the female runner. Wilder RP, OìConnor F, Herausgeber. BMR. 1996;6(1):37647.
20. Hale RW, Milne L. The elite athlete and exercise in pregnancy. Seminars in Perinatology. 1996;20(4):277684.
21. Heron N, Bigard X, Jones N. Union Cycliste Internationale (UCI) cycling pregnancy and postpartum guidance. Science & Sports. 2023;38(7):676682.
22. Holt EL, Holden AV. A risk-benefit analysis of maintaining an aerobic-endurance triathlon training program during pregnancy: A review. Science & Sports. 2018;33(5):e18169.
23. Huch R. Leistungssport in der Schwangerschaft. In: Keller E, Herausgeber. Frau im Leistungssport. Springer-Verlag; 1988. S. 85697.
24. Irani CR, Turner EHG, Rumps MV, Mulcahey MK. Recommendations for postpartum athletes returning to sport: the past, present, and future. The Physician and Sportsmedicine. 2024;52(6):533640.

25. Jackson T, Bostock EL, Hassan A, Greeves JP, Sale C, Elliott-Sale KJ. The Legacy of Pregnancy: Elite Athletes and Women in Arduous Occupations. Exercise and Sport Sciences Reviews. 2022;50(1):14ó24.
26. Kehler AK, Heinrich KM. A selective review of prenatal exercise guidelines since the 1950s until present: Written for women, health care professionals, and female athletes. Women and Birth. 2015;28(4):e93ó8.
27. Kimber ML, Meyer S, Mchugh TL, Thornton J, Khurana R, Sivak A, u. a. Health Outcomes after Pregnancy in Elite Athletes: A Systematic Review and Meta-analysis. Medicine & Science in Sports & Exercise. 2021;53(8):1739ó47.
28. Kramarz S, Lenzen-Schulte M. Leistungssport und Beckenboden ó Harninkontinenz ist hÊufig. GynÊkologie. 2023;56(10):738ó44.
29. LìHeveder A, Chan M, Mitra A, Kasaven L, Saso S, Prior T, u. a. Sports Obstetrics: Implications of Pregnancy in Elite Sportswomen, a Narrative Review. JCM. 2022;11(17):4977.
30. Leistungssport und Schwangerschaft ó aktuelle Æmpfehlungen und Gˇte der aktuellen Evidenzlage. SEMS. 2020; https://sems-journal.ch/9418.
31. Lumbers ER. Exercise in pregnancy: Physiological basis of exercise prescription for the pregnant woman. Journal of Science and Medicine in Sport. 2002;5(1):20ó31.
32. McGregor B, McGrath R, Young J, Nottle C. A scoping review of the experiences of elite female athletes concerning pregnancy and motherhood. Sport in Society. 2024;27(8):1221ó53.
33. McManis BG. Integrative Review of Exercise at Altitude during Pregnancy. IJERPH. 2. 2021;18(17):9272.
34. Noon ML, Hoch AZ. Challenges of the Pregnant Athlete and Low Back Pain: Current Sports Medicine Reports. 2012;11(1):43ó8.
35. Nose□Ogura S. Advancement in female sports medicine and preventive medicine. J of Obstet and Gynaecol. 2021;47(2):476ó85.
36. OìBrien M, Robertson A. Women and Sport. Scott Med J. 2010;55(2):25ó8.
37. Pasque CB. Women in Combat Sports. In: Wallace EA, Herausgeber. Combat Sports Medicine. London Springer; 2010. S. 135ó49.
38. Physical Activity and Exercise During Pregnancy and the Postpartum Period: ACOG Committee Opinion, Number 804. Obstetrics & Gynecology. 2020;135(4):e178ó88.
39. Pivarnik JM, Perkins CD, Moyerbrailean T. Athletes and Pregnancy: Clinical Obstetrics and Gynecology. 2003;46(2):403ó14.

40. Pivarnik JM, Szymanski LM, Conway MR. The Elite Athlete and Strenuous Exercise in Pregnancy. Clinical Obstetrics & Gynecology. 2016;59(3):6136́9.
41. Santos-Rocha R, Szumilewicz A. Fostering the social and physical challenges of female recreational or professional athletes in pregnancy and postpartum. BMC Pregnancy Childbirth. 2024;24(1):128, s12884-024-063126́6.
42. Schippert C, Legerlotz K. Schwangerschaft und Spitzensport. Leistungssport. 2023;2:336́ 6.
43. Schlegel P. From caution to guidance: a narrative review of CrossFit during pregnancy. Hum Mov. 2024;25(2):16́11.
44. Selman R, Early K, Battles B, Seidenburg M, Wendel E, Westerlund S. Maximizing Recovery in the Postpartum Period: A Timeline for Rehabilitation from Pregnancy through Return to Sport. International Journal of Sports Physical Therapy 2022;17(6). https://ijspt.scholasticahq.com/article/37863-maximizing-recovery-in-the-postpartum-period-a-timeline-for-rehabilitation-from-pregnancy-through-return-to-sport
45. Spowart L, McGannon KR. Motherhood and Sport: Collective Stories of Identity and Difference. 1. Edition. London: Routledge; 2022. https://www.taylorfrancis.com/books/9781003140757
46. Sulprizio M, L˘w R, Schulte-Frei B, JÊger L. Trainingsempfehlungen zum Sport in und nach der Schwangerschaft. In: Sulprizio M, Kleinert J, Herausgeber. Sport in der Schwangerschaft. Berlin, Heidelberg: Springer Berlin Heidelberg; 2016 S. 696́80. https://link.springer.com/10.1007/978-3-662-48760-0_7
47. Thein-Nissenbaum J. The postpartum triathlete. Physical Therapy in Sport. 2016;21:956́ 106.
48. Tighe BJ, Williams SL, Porter C, Hayman M. Barriers and enablers influencing female athlete return-to-sport postpartum: a scoping review. Br J Sports Med. 2023;57(22):14506́ 6.
49. Titova J, Davenport MH, Humphrys A, Hayman M. Barriers and enablers encountered by elite athletes during preconception and pregnancy: a mixed-methods systematic review. Br J Sports Med. 2024;bjsports-2024-108380.
50. Torres-Santos A, Avila GV, Garcia-Garro P. THE INFLUENCE OF PREGNANCY ON THE PHYSIOLOGY OF ELITE SPORTSWOMEN: A NARRATIVE REVIEW. RICCAFD. 2024;13(1):1386́55.
51. Warren MP, Shantha S. The female athlete. Best Practice & Research Clinical Endocrinology & Metabolism. 2000;14(1):376́53.

52. Wieloch N, Klostermann A, Kimmich N, Sp˘rri J, Scherr J. Sport and exercise recommendations for pregnant athletes: a systematic scoping review. BMJ Open Sport Exerc Med. 2022;8(4):e001395.
53. Woodroffe L, Slayman T, Paulson A, Kruse N, Mancuso A, Hall M. Return to Running for Postpartum Elite and Subelite Athletes. Sports Health: A Multidisciplinary Approach. 2024;19417381241256973.
54. Wowdzia JB, Mchugh TL, Thornton J, Sivak A, Mottola MF, Davenport MH. Elite Athletes and Pregnancy Outcomes: A Systematic Review and Meta-analysis. Medicine & Science in Sports & Exercise. 2021;53(3):534ó642.
55. Zavorsky GS, Longo LD. Last Word on Viewpoint: Are there valid concerns for completing a marathon at 39 weeks of pregnancy? Journal of Applied Physiology. 2012;113(7):1167ó1167.
